# Human lung-cancer-cell radioresistance investigated through 2D network topology

**DOI:** 10.1038/s41598-022-17018-0

**Published:** 2022-07-28

**Authors:** Luca Tirinato, Valentina Onesto, Daniel Garcia-Calderon, Francesca Pagliari, Maria-Francesca Spadea, Joao Seco, Francesco Gentile

**Affiliations:** 1 Department of Experimental and Clinical Medicine, Nanotechnology Research Center, University of Magna Graecia, 88100 Catanzaro, Italy; 2 Division of Biomedical Physics in Radiation Oncology, DKFZ - German Cancer Research Center, Heidelberg, Germany; 3 Department of Physics and Astronomy, Heidelberg University, Heidelberg, Germany; 4 Department of Experimental and Clinical Medicine, University of Magna Graecia, 88100 Catanzaro, Italy

**Keywords:** Oncology, Biophysical methods, Imaging, Microscopy, Sensors and probes, Image processing, Network topology, Cancer, Cancer prevention, Cancer therapy, Lung cancer

## Abstract

Radiation therapy (RT) is now considered to be a main component of cancer therapy, alongside surgery, chemotherapy and monoclonal antibody-based immunotherapy. In RT, cancer tissues are exposed to ionizing radiation causing the death of malignant cells and favoring cancer regression. However, the efficiency of RT may be hampered by cell-radioresistance (RR)—that is a feature of tumor cells of withstanding RT. To improve the RT performance, it is decisive developing methods that can help to quantify cell sensitivity to radiation. In acknowledgment of the fact that none of the existing methods to assess RR are based on cell graphs topology, in this work we have examined how 2D cell networks, within a single colony, from different human lung cancer lines (H460, A549 and Calu-1) behave in response to doses of ionizing radiation ranging from 0 to 8 Gy. We measured the structure of resulting cell-graphs using well-assessed networks-analysis metrics, such as the clustering coefficient (*cc*), the characteristic path length (*cpl*), and the small world coefficient (SW). Findings of the work illustrate that the clustering characteristics of cell-networks show a marked sensitivity to the dose and cell line. Higher-than-one values of SW coefficient, clue of a discontinuous and inhomogeneous cell spatial layout, are associated to elevated levels of radiation and to a lower radio-resistance of the treated cell line. Results of the work suggest that topology could be used as a quantitative parameter to assess the cell radio-resistance and measure the performance of cancer radiotherapy.

## Introduction

Radiation therapy (RT) is a method of cancer treatment based on the exposition of cancer tissues and organs to some kind of ionizing radiation that damages the genetic material of cells, inhibits their functions, including proliferation and growth, and causes in the end their death^1,2^. In the current clinical practice, approximately half of the patients diagnosed with cancer receive RT alone or as part of their treatment, in combination with chemotherapy and surgery^3^. The term RT in fact designates a number of different variations of the technique such as, to cite a few, external beam radiotherapy^4,5^ (EBR) or internal radioisotope therapy^6–8^ (RIT). In the former, radiation beams are guided from the external of the body to the target tissue, and in the latter therapeutic radioisotopes are positioned through the systemic circulation in close proximity of cancer cells for local cancer therapy. Remarkably, both techniques can take benefit from recent technological evolutions and achievements, such as modern biomedical imaging^9–11^ and nanomedicine and nanotechnology^12,13^. While radiotherapy offers promise to treat cancer, nevertheless some inherent limitations can reduce the effectiveness of the technique. *Radioresistance* (RR) is a process which indicates an innate or acquired ability of tumor cells to resist to treatments following a variety of different mechanisms such as, among others, strong DNA damage repair ability, autophagy, adaptation to hypoxia and presence of cancer stem cells, associated with changes in the tumor microenvironment^14,15^. RR is a major factor leading to RT failure.

The effectiveness of radiation treatments and the degree of RR depend on five main factors (5 R’s), derived from almost a century of research in radiation biology. These are: (1) repair, describing the ability to repair non-lethal DNA damages; (2) repopulation after radiation, which is different in early and late responding tissues; (3) redistribution of cells along the cell cycle; (4) reoxygenation of surviving cells; (5) radiosensitivity. While the first 4 factors were described initially by Withers^16^, the last one was added by Steel and collaborators^17^ upon several observations that tumor radiation response was strongly dependent on an intrinsic cellular radiosensitivity. These 5 factors can work in opposite directions depending on two main parameters, i.e. the type of tumor and the radiation type and how it is delivered. From a clinical point of view, the most common plan used to treat patients with photon radiotherapy is based on fractionated treatments, in which a dose of around 2 Gy/day is given for 5 days in a week, commonly for a period of between 4 and 8 weeks. This aims at maximizing the noxious effects on malignant cells, while reducing the damages to healthy tissues. In fact, this strategy increases the cell killing by redistributing and reoxygenating resistant cancer cells into more sensitive types. However, other parameters, such as cellular damage repair and repopulation, can favor cancer cell regrowth after each radiation fraction.

It is of the utmost importance to develop and consolidate techniques and methods that can assess the sensitivity of cells to internal or external radiation fields. State-of-the-art methods used to test cell RR and radio-sensitivity include, but are not limited to, cell proliferation assays^18,19^, neutral comet assay^20^, gamma-H2AX immunofluorescence staining^21,22^, and clone formation assay^23^. This latter is widely and routinely used in radiation biology because it allows to correlate cell radiosensitivity to survival and to clinical responses to RT of tumor cell lines and patient-derived tumor cells^24,25^.

The evaluation of a diverse cancer cell radiosensitivity has been recently accomplished through the identification of their morphological characteristics. In reference^26^, Gray and colleagues reported on some morphological changes that canine and human mammary cancer cell lines experience with the acquisition of RR in 2D cultures. In reference^27^, Sham and colleagues showed that, upon exposition to gamma ray radiation, mouse breast cancer (EMT6) cells underwent a series of morphological changes, including a glandular pattern, a vacuolated cell plasma, pleomorphic nuclei and an enlarged size. In another reported study^28^, Yasser and collaborators observed an altered cell morphology in RR oral cancer sublines, i.e. 50Gy-UPCI:SCC029B and 70Gy-UPCI:SCC029B, subjected to 2 Gy fractionated ionizing radiation (FIR) compared to the parental UPCI:SCC029B cell line. This treatment induced morphological changes resulting in a significant increase of the filopodia number in RR cells.

The clonogenic assay is considered as the gold standard for studying the radiation response of cancer cells, where the number of surviving colonies for each radiation dose are counted. However, several parameters can affect profoundly the results, such as cell type, doubling times, cell density, cellular cooperation, and cell nutrient availability. In the clonogenic assay performed in radiation biology studies, each colony is still considered as a “black box”, as the properties of the cancer cells within each survival colony are commonly ignored and the colonies are considered as single entities without looking at the intrinsic cellular variability. In this respect, it has been shown that single primary and cancer cells can give rise to different types of colonies (defined holoclones, meroclones and paraclones) based on their proliferative abilities and stemness potential^29–31^.

In this work, we have examined how the topological characteristics of lung cancer cells within the surviving colonies changed with increasing doses of ionizing radiation in 2D culture conditions. Therefore, we focused on the variability inside the colonies based on the physical features of the cells rather than on their proliferative capacity. While this study has been demonstrated in 2D systems, nonetheless results of the work and the method can be extended to more complex, but also more informative, 3D geometries, in which cells do not suffer from limited cellular cooperation^32^. These aspects are discussed in detail later in the paper.

Cultured lung cancer (H460, A549 and Calu-1) cells were exposed to X-rays at different doses ranging from 8 to 8 Gy, producing a clonogenic survival curve. After exposure, we imaged each colony of cells using fluorescence microscopy and measured the topological characteristics of the cells within the colony using networks science.

These specific lung cancer cell lines were chosen on the ground of arguments such as: (1) they are derived from the same organ and are similar in type (non-small cell lung carcinoma), but at the same time exhibit differences, that make them ideal sample-cells for a comparative analysis; (2) they are very well characterized in terms of doubling times, among other characteristics, that are conveniently reported in the methods of the work.

**Figure 1:**
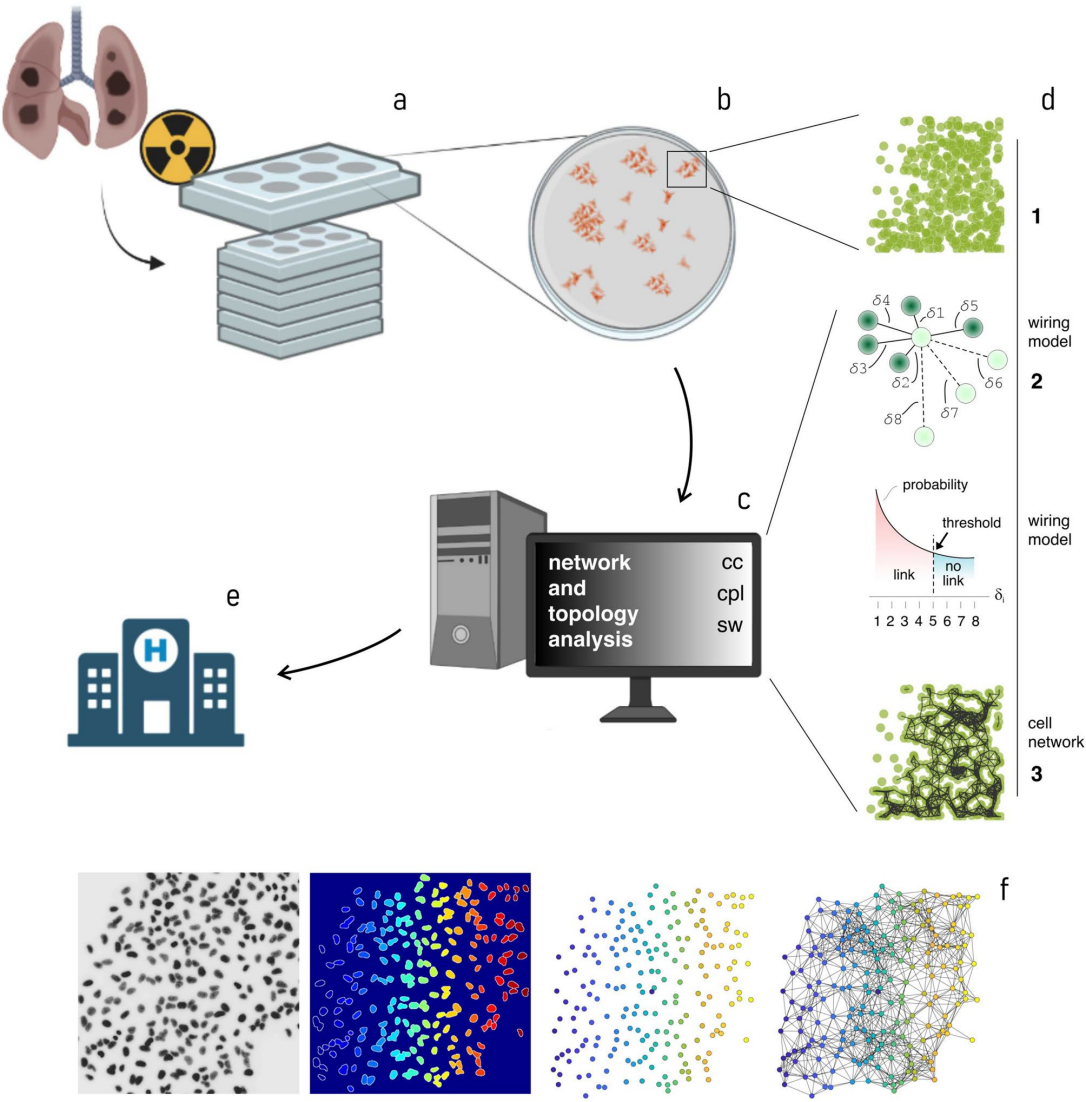
Flowchart of the different steps of the study. Different lung cancer cells (H460, A549 and Calu-1) were cultured on a 6-multi-well plate and exposed to an ionizing radiation in the 0–8 Gy range (**a**). Cells were then images using fluorescence microscopy (**b**). The acquired images were then passed to a computer for processing and data analysis (**c**). The data analysis consisted in well-assessed technique of network science, including: routing the nodes, building the associated cell graphs, and extraction of network parameters such as the clustering coefficient, the characteristic path length, and the small world coefficient (**d**). The topological parameters determined through the analysis where then correlated to the biological characteristics of cells and the radiation dose. This procedure and the association between the topology of cancer cell graphs, the radiation, and the cell characteristics, can be possibly used in clinical medicine as a method for determining the radioresistance of cells and the performance of radiotherapy (**e**). Part (**f**) of the figure illustrates how graphs were generated out of cells. The originating fluorescent cell images were segmented and isolated by a watershed transformation and a distance transform. The process enabled to split out separated, non-touching regions in each image that were thus identified as the cell-centers. Cell centers were then connected using the Waxman’s model: the Waxman algorithm finds the probability of two nodes being linked as a negative exponential function of their distance. (Partially created with BioRender.com).

Then, for each dose and cell line, we determined the average number of links per node (*k*), the average values of clustering coefficient (*cc*), characteristic path length (*cpl*) and small world coefficient (SW) of graphs of cells resulting from the interaction of the cultures with the external radiation. The flowchart of the separate steps of the process is reported in Fig. 1. We then put in relation the topological measures of cell networks with the dose and the cell morphological characteristics. We show that high value of SW coefficient greater than one are associated with high levels of radiation and to a lower radio-resistance of the treated cell lines.

## Results

### Cell-adhesion at different radiation doses

**Figure 2:**
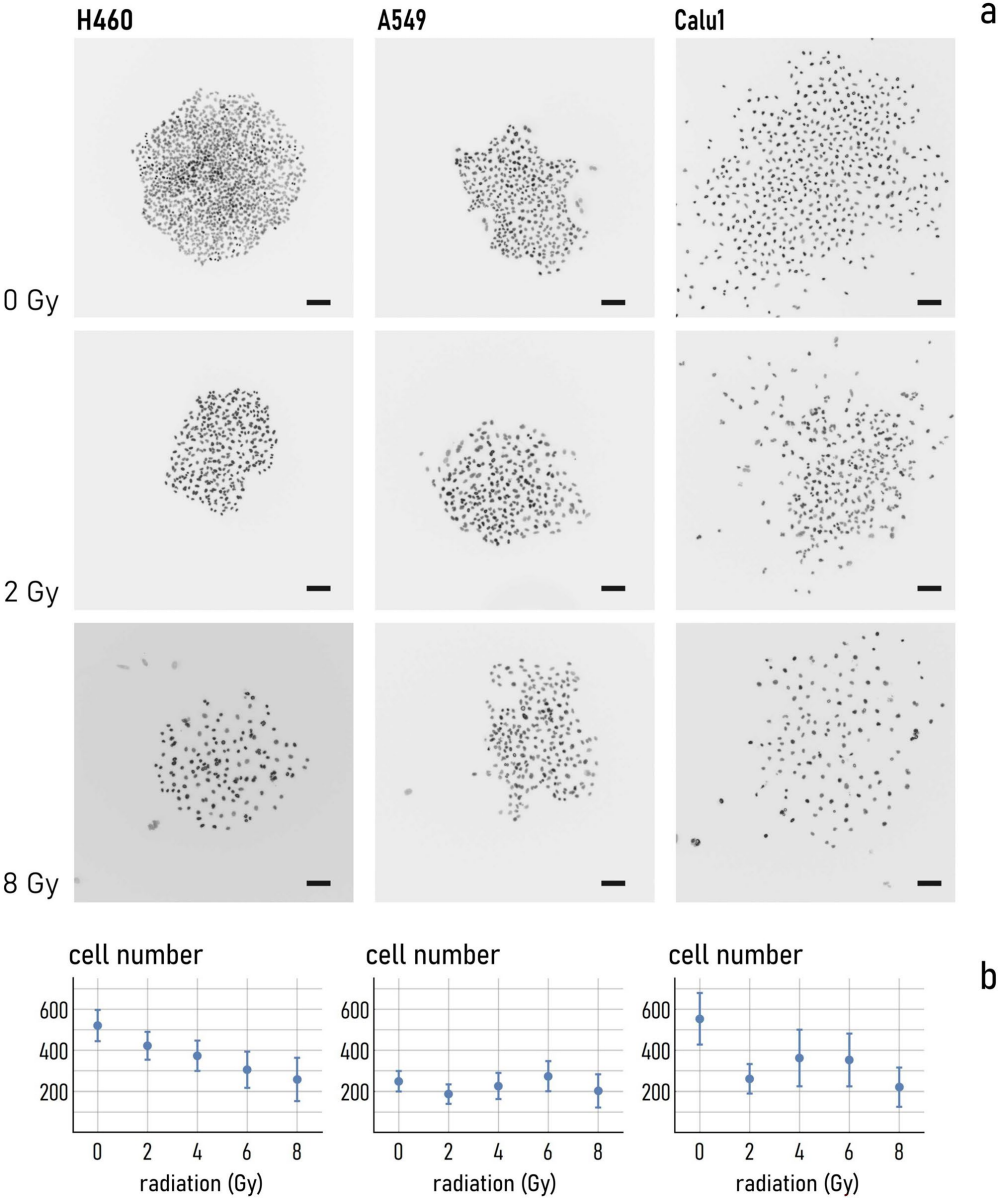
Representative fluorescent images of H460, A549 and Calu-1 cells taken with a 10X objective at 0 Gy and at different values of the irradiation dose: 2, 8 Gy (**a**). Number of H460, A549 and Calu-1 cells on the substrate evaluated at different levels of the dose (**b**). For all images the scale bar is 100 µm.

The number of cells initially seeded on the plates varied at densities proportional to the increasing doses to account for the delayed growth ability investigated. The values of incubation time and cell number that we used in the experiments were determined using a standard linear-quadratic model to have a quasi-constant cell density at the final time of the experiment^33^. Then, at the prescribed times, cells were fixed, stained with DAPI, and imaged using an inverted fluorescence microscope equipped with 40×/10× objectives, as described in the methods. We used custom-made data analysis algorithms^34^ to examine the fluorescence images and calculate the number of cells adhering to each plate at the end of the experiments. Figure 2a shows fluorescence images of not-irradiated cell nuclei and nuclei after exposition to low (2 Gy) and high (8 Gy) radiation doses. Direct comparison between images indicates that the cell density on the substrates varied moving from 0 to 8 Gy to an extent that depends on the considered cell line. For H460 cells, the average number of cells (*n*) steadily decreases from 500 for 0 Gy to 250 for 8 Gy (Fig. 2b). For A549 cells, *n* oscillates in the narrow 200–280 interval for the considered range of doses. For Calu-1 cell line, *n* is equal to approximately 550 at 0 Gy, it falls to 250 for a 2 Gy dose, and then it fluctuates from 250 to 200 moving from 2 to 8 Gy, passing through the $n\sim 380$ value at 4 and 6 Gy. Thus, as regarding cell-adhesion of the considered samples, H460 cells show a higher sensitivity to the radiation dose, with a 50% variation measured in the 0–8 Gy range. Conversely, except for the 0—Gy dose for Calu-1, the A549 and Calu-1 cell lines exhibit a constant behavior and are only moderately influenced by the external radiation. In these experiments, all cell lines were seeded the day before of the irradiation with a density determined in reason of their different growth rates and doubling times. This allowed to have, at the time of irradiation sub-confluent cultures, comparable cell densities, and high sample size, necessary to assure statistical significance of the analysis.

### Cell-network topology within a colony at different radiation doses

**Figure 3:**
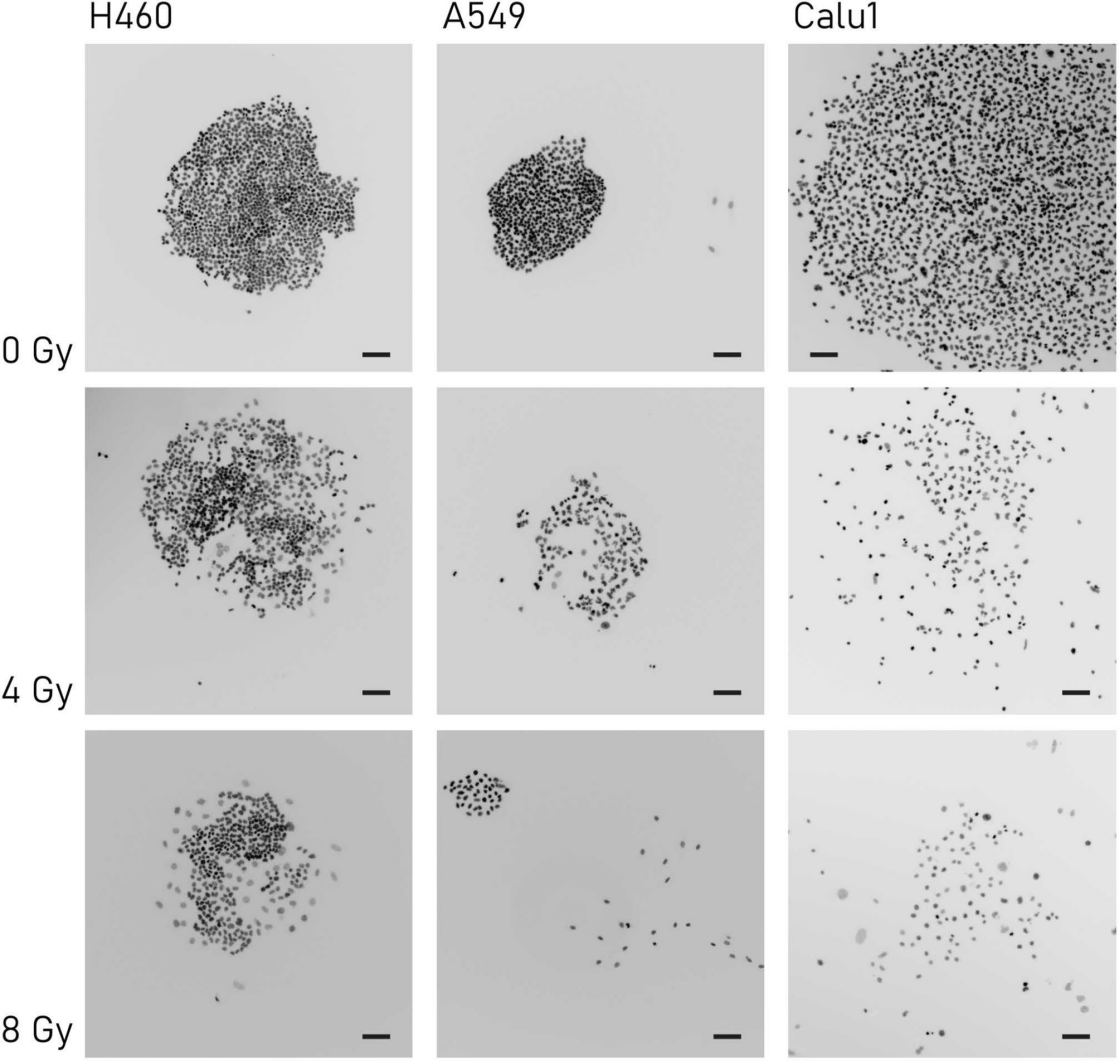
The image shows for different cell lines (H460, A549 and Calu-1 cells) and different values of the irradiation dose (0, 4, 8 Gy) the cell distribution and spatial layout on the substrate. Visual inspection of cell distribution suggests that the topology of cell groups is influenced by the irradiation dose. The scale bar is 100 µm.

Visual inspection of fluorescence images of cells taken at different doses indicates that the ionizing radiation influences likewise the spatial layout of those cells on the substrate. Images in Fig. 3 illustrate that the clustering properties of cells varies significantly moving from 0 to 8 Gy. In the first case (0 Gy), the distribution of cells on the surface is homogeneous, while in the second case (8 Gy) cells are packed irregularly, with cells occasionally clustering into small structures with higher density than that in the surrounding regions. Notice that Figs. 2a and 3 are similar: they both show the distribution and organization of cells in a colony at different radiation doses as a function of cell type. Nevertheless, while Fig. 2a is more focused on the number of cells in a colony, Fig. 3 is meant to illustrate how the topology characteristics of cells vary as a function of the radiation dose. We used topological metrics to describe quantitatively the features of the networks formed by the cells under different radiation conditions. To do this, images of cells were segmented using the algorithms described in the Methods of the paper. Then, after locating all cell centers and contours, we associated to each image a network, generated by linking cell-centers through the canonical Waxman algorithm (Fig. 1f). The Waxman algorithm makes a decision on whether two elements of a system are connected basing on their distance: the smaller the interspace between elements, the higher the likelihood that these elements are joined by a link^35^. Cast in mathematical terms, nodes *a *and *b* are connected if $\alpha exp\left( { - \beta d_{ab} /l} \right) > p$, where *d*_*ab*_ is the Euclidean distance between *a* and *b*, *l* is a reference length, *α* and *β* are model parameters, and *p* is a cut-off probability. Following this procedure, we derived for each of the images acquired for the considered values of radiation dose and cell lines the corresponding networks, some of which are reported in a separate Supporting Information Fig. 1 for the sake of illustration. The number of links per node (degrees) and the degree distribution, i.e. the fraction of nodes with a certain degree, are some of the parameters used to characterize the networks.

**Figure 4:**
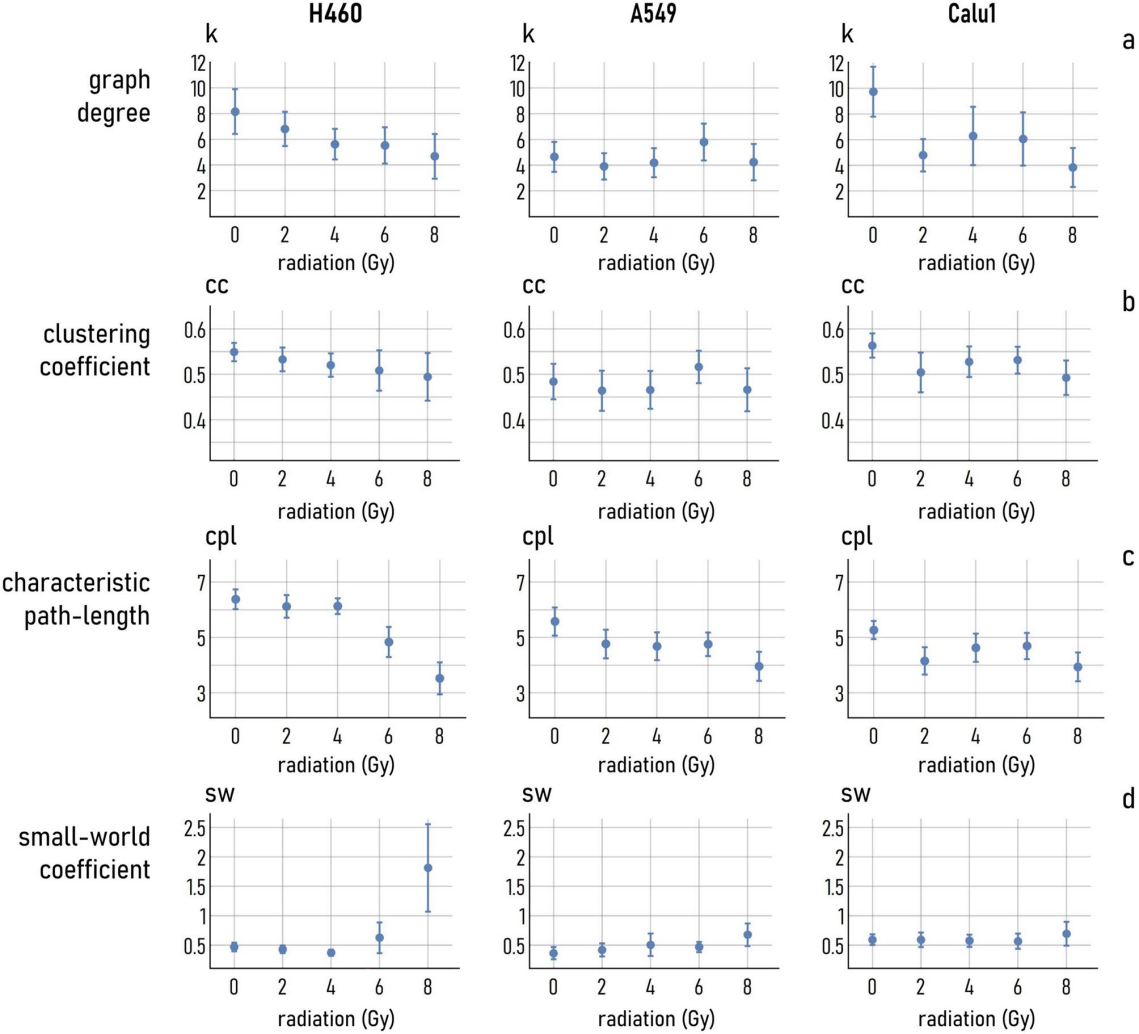
The degree of a graph *k* (**a**), clustering coefficient *cc* (**b**), characteristic path length *cpl* (**c**) and small-world coefficient SW (**d**) of the cell-graphs obtained by linking cell centers through the Waxman algorithm are reported.

We report in Fig. 4 the values *cc*, *cpl*, and SW, determined for cell-graphs associated to different doses and cell lines—i.e. the most significant topology parameters that are used to characterize networks. The network analysis was conducted setting a cut-off probability $p = 0.95$. The robustness of the results to changes of *p* are discussed later in the paper.

The network degree (*k*) is the average number of links per node in a graph. We observe that, in the 0–8 Gy interval, *k*: (1) smoothly passes from $k\sim 8$ to $k\sim 4.2$ for H460 cells; (2) it is stable around the value 4 for A549 cells; (3) it shifts from $k\sim 10$ to $k\sim 4.2$ in the low radiation range and oscillates afterwards around 4 for the Calu1 cell line.

The clustering coefficient (*cc*) is the proportion of links existing among the nodes enclosing an element of the grid, to the total number of connections that can be possibly established among those nodes, averaged all over the elements of the network. It is a measure of graph connectivity and ranges between 0 and 1. We observe that the trend of variation of *cc* with the dose is similar to the trend measured for *k*. For the H460 cell line, *cc* varies linearly from $cc\sim 0.55$ for 0 Gy, to $cc\sim 0.5$ for 8 Gy. For A549 and Calu1 cells, the clustering coefficient fluctuates around $cc\sim 0.48$ and $cc\sim 0.51$, respectively. In any case, the variations are below 0.05 and the clustering coefficient can be considered stable in the considered range of ionizing radiation doses.

The characteristic path length (*cpl*) is the average shortest path among elements in a grid. For the H460 cell line, *cpl* is seemingly constant and equal to 6.1 in the 0–4 Gy range, then it starts to decrease and assumes the value $cpl\sim 3.3$ at 8 Gy. The variations of *cpl *for A549 and the Calu-1 cells are less relevant, with *cpl* changing from $\sim 5.5$ to $\sim 4$, and from $\sim 5$ to $\sim 4$, for the former and the latter of these cell lines respectively in the 0–8 Gy interval.

The small world coefficient (SW) is determined as a combination of *cc* and *cpl *(methods). It is a measure of the average connectivity (*cc*) and average proximity (*cpl*) between the elements of a network *G*, relative to an Erdos–Renyi random graph with the same size of *G* ^36^. The SW indicates to which extent elements of a network cluster into groups^37–40^, which in turn correlates to the enhanced capability of that network of transporting information or signals^34,41–46^. The values of small-world-ness that we have found for H460 cells is quasi constant in the 0–6 Gy interval with small deviations around 0.5, then it soars to ${\text{SW}}\sim 1.8$ when the radiation dose is set as 8 Gy. For A549 cells, the SW faintly increases with the dose, moving from ${\text{SW}}\sim 0.4$ at 0 Gy to ${\text{SW}}\sim 0.7$ at 8 Gy. For Calu-1 cells, the values of *sw* are only limitedly influenced by the dose, with a measured value of ${\text{SW}}\sim 0.5$ at 0 Gy and ${\text{SW}}\sim 0.7$ at 8 Gy.

### Cell-morphology within a colony at different radiation doses

**Figure 5:**
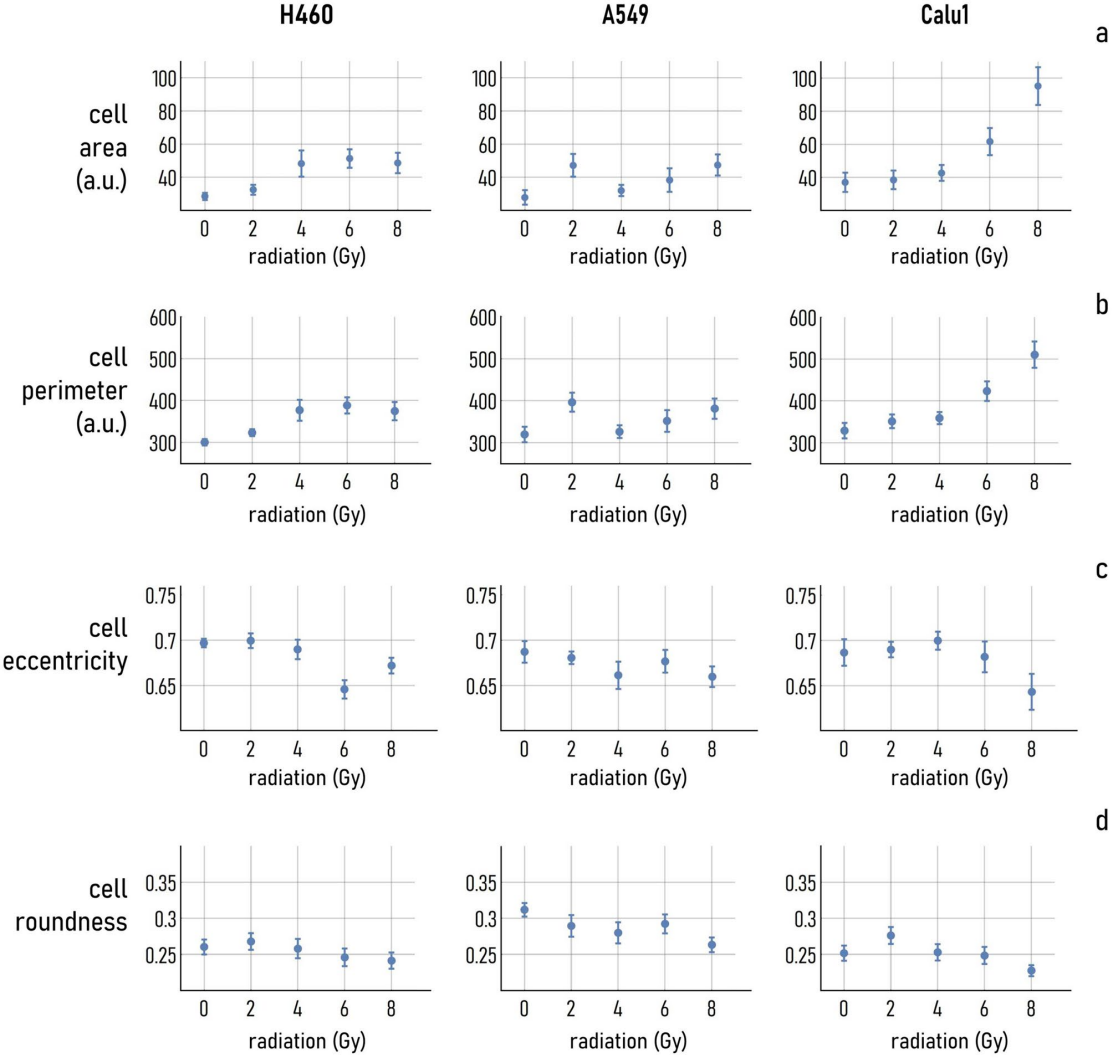
Cell morphological parameters extracted from the three cell lines (H460, A549, Calu-1) as a function of the external radiation in Gy. Specifically, we have determined the cell area (**a**), perimeter (**b**), eccentricity (**c**) and roundness (**d**) for the 0, 2, 4, 6, 8 Gy radiation doses.

Fluorescence images of cells taken at a higher (40×) magnification indicate that the morphological characteristics of cells are influenced by the radiation dose to some extent and in any case in a different fashion than topology (Supporting Information Fig. 2). Of the considered cell lines, Calu-1 cell morphology seems to be more sensitive to the dose. Using image analysis algorithms (described in Methods), we extract for each image different metrics that describe the morphology of the cells quantitatively, i.e. the average area, perimeter, eccentricity and roundness. Of these estimates, the cell area and perimeter are expressed in pixels. The eccentricity is the ratio of the distance between the foci of the cell ellipse and its major axis length; it is zero for a circle and is greater than zero for an ellipse. The roundness is the ratio between 4*π* times the area of the cell and its perimeter squared, thus, for a perfect circle, the roundness is 1. Both the eccentricity and roundness are dimensionless. For H460 cells, the average cell area varies from about 20 pixels for untreated cells (0 Gy) and low radiation dose (2 Gy) to about 50 pixels for higher radiation doses (4, 6, 8 Gy). Similarly, the cell perimeters raise from about 300 pixels, measured at 0 Gy, to nearly 400 pixels, measured for doses higher than 4 Gy (Fig. 5). Both the area and the perimeter attain to a steady state value for dose values higher than 4 Gy. The eccentricity and roundness seem to be affected to a lower extent by the dose. The eccentricity varies of less than 5 percentage points in the 0–8 Gy range, and the roundness of less than $1$ percentage point in the same interval.

Results of the analysis indicate that the morphology of A549 cells is only marginally influenced by the dose, with average values of area and perimeter that start to vary linearly only after 4 Gy, with a slope of $\sim 2.5$ pixels/Gy for the area, and $\sim 15$ pixels/Gy for the perimeter. In the same way, the cell eccentricity oscillates between $\sim 0.68$ and $\sim 0.66$, and $\sim 0.31$ and $\sim 0.26$, for a dose moving from 0 to 8 Gy (Fig. 5).

Differently from the H460 and A549 cell lines, the shape of Calu-1 cells is significantly influenced by the radiation dose. The growth of the cell area and perimeter is seemingly exponential, with a final area value (perimeter) of $\sim 98$ ($\sim 510$) pixels, calculated at 8 Gy, compared to an initial area value of (perimeter) of $\sim 36$ ($\sim 315$) pixels determined at 0 Gy. Higher radiation doses also cause a change in the aspect ratio of the cells, that become more circular in shape at 8 Gy with an eccentricity less than 0.65, compared to an eccentricity greater than 0.70 determined at 0 Gy. At odds with the eccentricity, the roundness is limitedly affected by the dose, with a variation of $\sim 0.02$ points, from $\sim 0.25$ to $\sim 0.28$, for a dose value changing from 0 to 8 Gy.

### Statistical analysis of the cell topology and morphology at varying radiation doses

**Figure 6:**
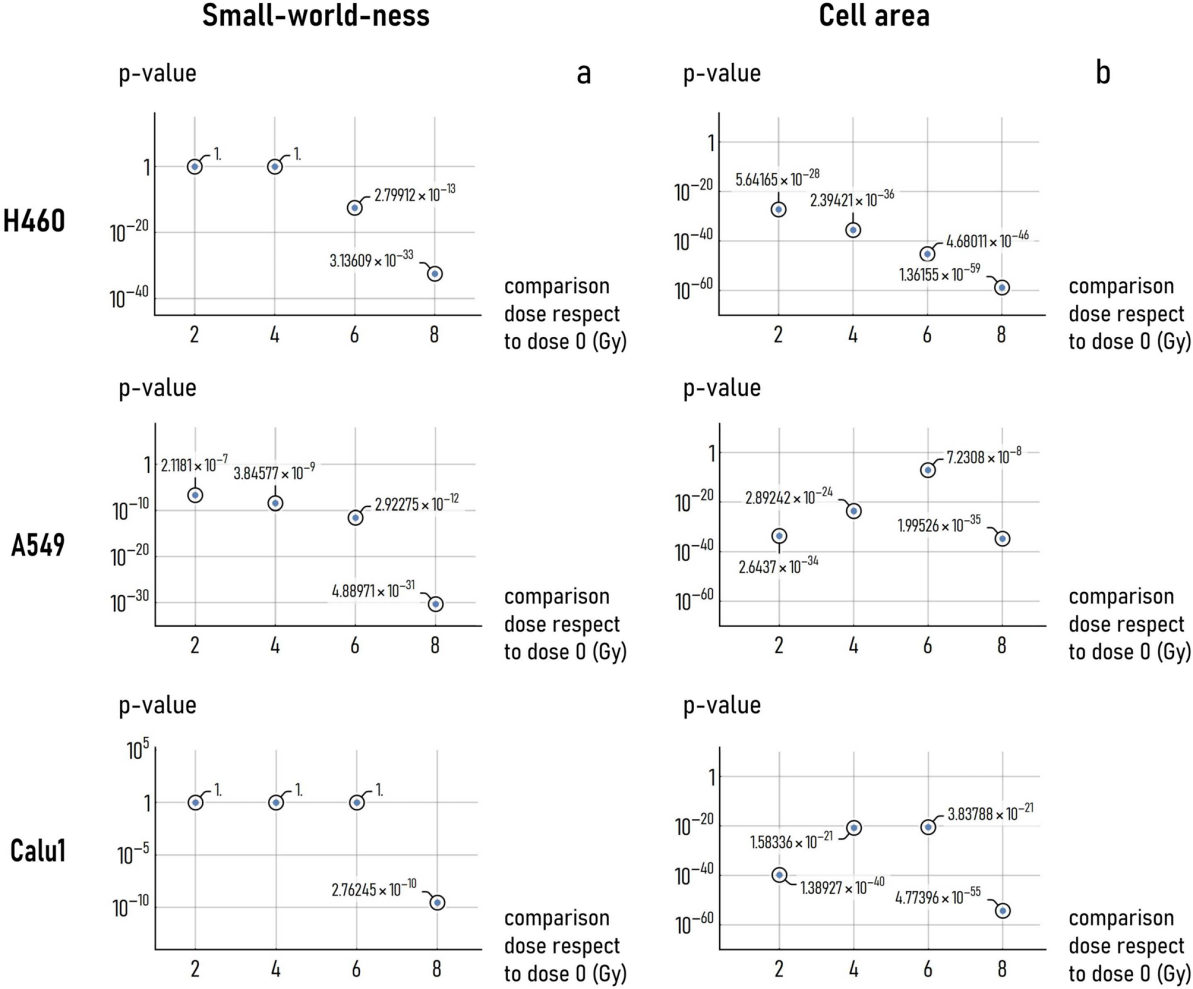
Statistical analysis of cell-graphs topology and morphology. Diagrams in (**a**) show the *p*-value associated to a Student’s T-test comparison statistics between the SW coefficient derived for non-exposed cells (0 Gy) and cells exposed to 2, 4, 6 and 8 Gy dose. Diagrams in (**b**) show the *p*-value associated to a Student’s *t*-test comparison statistics between the area determined for non-treated cells (0 Gy) and cells exposed to 2, 4, 6 and 8 Gy dose. The smaller the *p*-value, the larger the statistical significance of the difference between the topology/morphological parameters measured for cells subjected or not to external radiation.

We used a two sample Student’s t test to examine whether the cell topology and morphology are significantly affected by the radiation dose. For each of the considered cell lines, we compared the distributions of values of small-world-ness obtained at 0 Gy with those calculated at higher radiation doses, i.e. 2, 4, 6 and 8 Gy (Fig. 6a). In the same way, we compared the cell area calculated for a 0 Gy exposition dose, to the values determined upon exposure to higher doses, up to 8 Gy (Fig. 6b). Given that *H*_0_ (null-hypothesis) assumes that the samples are extracted from the same population, we tested our hypothesis with 95% confidence interval (i.e. $= 0.05$). The diagrams in Fig. 6a indicate that the topology of the H460 cell networks is influenced by the ionizing radiation in the high dose range (*p*-value $< 0.05$ for a dose greater or equal than 6 Gy), and that for A549 cells the SW is statistically different from that calculated for the not-exposed samples for any considered dose. Conversely, Calu-1 cells seem to resist (i.e. show no variation) to the radiation dose up to 6 Gy (*p*-value $\gg 0.05$), and only at 8 Gy the graphs formed by those cells are topologically different from the networks generated by routing the same cells in absence of external radiation (*p*-value $< 0.05$) (Fig. 6a). As regarding cell morphology: differently from topology, the morphology is statistically influenced by the external radiation for any considered dose and cell line. Thus, the topology is less sensitive than morphology to the ionizing radiation dose.

## Discussion and conclusions

In this study, we have verified the topological and morphological characteristics of three different lung cancer cell lines against an external controlled photon radiation. Experiments conducted in previous reports^47–50^ have shown that, of these, the H460 cells are more radio-sensitive compared to the A549 or Calu-1 cell lines (*Methods*). Remarkably, results of the work obtained by alternative techniques are consistent with this existing biological description of cells RR.

In this experimental setup, where the radiation dose was varied in the 0–8 Gy interval, we found that the most relevant parameter describing the cell-network topology, i.e. SW, deviates significantly from the ground-value measured in absence of radiation (0 Gy) for any dose greater or equal than $t^{H460} = 6$ Gy for H460 cells. This threshold shifts to $t^{A549} = 2$ Gy for A549 cells, and bounces to a $t^{Calu1} = 8$ Gy high for Calu-1 cell line. Moreover, we found that the mean SW of the cell graphs measured at the maximum available dose (8 Gy) is equal to ${\text{SW}}\sim 1.82$, ${\text{SW}}\sim 0.67$ and ${\text{SW}}\sim 0.70$, for H460, A549 and Calu-1 cell lines, respectively. Comparing these values to the coefficients measured at 0 Gy, we found that the small-world-ness enhancement factor from 0 to 8 Gy is $E^{H460} = 3.81$, $E^{A549} = 1.85$ and $E^{Calu1} = 1.17$, for the considered cell lines. Moving from 0 to 8 Gy, the proportion between the clustering coefficient and average path length in the grids increases of nearly 4 times for H460 cells, of nearly 2 times for A549 cells and is about 1 for Calu-1 cells. Thus, the layout of cancer cells on a common culture substrate is influenced by the dose and cell characteristics. As already known, the larger the dose and the lower the cell radio-resistance, but the small-world attributes of groups of cells subjected to radiation appears to be more relevant.

The SW and enhancement factors that we have determined for the considered cell lines, and the *p*-values indicating the statistical distance among cell-graph-topologies measured at different values of the radiation dose, are conditional on the values of the Waxman model’s parameters that we have used to route cell nodes. To examine the sensitivity of SW of cell networks to changes of the values of the model parameters we performed additional tests. We generated cell-graphs from the same data using two additional, different values of the Waxman cut-off probability, $p = 0.9$ and $p = 0.98$, that add to the $p = 0.95$ figure already used in the work. The SW determined for these graphs are reported in a separate Supporting Information Fig. 3a. The associated *p*-values measuring the difference between sample means determined at different values of the radiation dose are reported in the Supporting Information Fig. 3b. Diagrams indicate that the trend observed for the $p = 0.95$ case is also maintained for the other two cut-off probability values, albeit coefficients may marginally vary in their absolute measures. For $p = 0.90$, the average SW of the cell graphs measured at 8 Gy is equal to ${\text{sw}}\sim 1.48$, ${\text{SW}}\sim 0.73$ and ${\text{sw}}\sim 0.94$, for the H460, A549 and Calu-1 cell lines, yielding small-world enhancement factors of $E^{H460} = 5.58$, $E^{A549} = 2.91$ and $E^{Calu1} = 2.35$, respectively. For $p = 0.98$, the mean *sw* of the cell graphs measured at 8 Gy is equal to ${\text{SW}}\sim 1.32$, ${\text{SW}}\sim 0.70$ and ${\text{SW}}\sim 0.65$, for the considered cell lines, leading small-world amplifications of $E^{H460} = 1.75$, $E^{A549} = 1.26$ and $E^{Calu1} = 1$.

In any case, larger doses result in high values of small-world-ness, and the more we move from high to low radio-resistant cells, the larger this effect. Cell network and statistical data analysis indicate that topology is a parameter that should be considered to evaluate the cancer cell sensitivity to external radiation fields. Moreover, topology might allow to unveil new features, ignored so far, of cancer radioresistant cells within those colonies considered as mere “black boxes” for too much long time.

Findings of this work have some remarkable consequences:

1. This procedure might be used as a new assay to test in vitro cancer radio-resistance where patient-derived cancer cells^51^ can be easily growth in 2D culture and followed over time upon exposure to different ionizing radiations (like photons, protons and heavy ions). The SW coefficient (topology) could be used as a metrics to assess cancer RR and/or sensitivity. The topological measures determined for a particular cell sample, dose and configuration should be then interpreted in the context of a database of known values that will be completed over time with convenient test-campaigns aimed to improve reliability, repeatability and accuracy as well as optimize the method performance.
2. Cell SW coefficient and topology could be both used to evaluate the status of living tissues subjected to ionizing radiation. This because, biological radiation injury is a major issue in biomedical imaging. The extent of the radiation damage occurs in various forms, depending on the ionizing radiation involved, the portion of the body exposed, the duration as well as the total amount of the dose absorbed^52^. A method based on topology can possibly detect earlier the cell/tissue damage signs caused by ionizing radiation, and provide to the physicians, biologists, and biomedical engineers with the support necessary to assess the risk and health effects of biomedical imaging.
3. The methodology here reported might be also purposely adapted and translated to investigate and unveil the functional clonogenic heterogeneity at the base of the RR.

However, the method that we have conceived and illustrated in this study has some limitations. The first and foremost, is that it has been applied to simple 2D systems of cells, while more complex 3D systems represent a better physiological condition in which cells do not suffer from limited cellular cooperation^32^. The opposition between 2 and 3D systems deserves to be commented even further. The preparation of 2D biological samples and colonies, and their measurement, is simpler compared to the analysis of 3D in vitro models. However, it is widely accepted that 3D cell culture and models are a more accurate representation of how cells grow or are affected by external factors, such as ionizing radiation^53^. The reasons why 2D models are still largely used–and why ourselves have analyzed bi-dimensional colonies–include: (1) low cost, (2) established technology and protocols, (3) existence of a great body of comparative literature, case studies and reports, (4) the measurement and inspection of cells is easy or comparatively easier than in 3D models. Conversely, 3D cell tissue models are biomimetic, physiologically relevant and predictive–in that they can replicate more faithfully the diffusion constraints usually found in the body that govern the exchange of nutrients, oxygen, signals and waste products among cells. 3D cultures and models also show a higher degree of structural complexity and retain a homeostasis for longer. Focusing on the particular theme of radiotherapy and cell radio-resistance: 3D culture systems and xenograft transplants, as in vitro and in vivo models respectively, better mimic the biochemical and biophysical properties of a tumor mass. In these systems, cell-to-cell interactions in a 3D environment, as well as cell architecture and polarity are important parameters that have to be considered when analyzing the topological features of the irradiated cells. The main challenges to face to extend our reported method to 3D systems are are high cost, moderate throughput, need of more complicated optical/measurement setup.

The method that we have developed necessitates the identification of the cell centers to build correctly the networks associated to the originating systems. While the information about 2D cell colonies is easily accessible and requires simple, low cost microscopy or fluorescence microscopy, determining the topology of 3D systems of cells such as 3D models and xenograft tumors may be challenging. This is due to the close packing and the spatial organization of cells that in 3D culture models cause many of the cells to be internal in the system, masked by other cells and not suited to direct optical inspection of the sample. In these respects, biomedical imaging techniques reveal complex structures organized over multiple scales, located deep inside the sample, that are otherwise difficult to decipher^54^. However, traditional imaging techniques, such as computer tomography (CT) scan, positron emission tomography (PET) scan, ultrasound imaging and sonography, magnetic resonance imaging (MRI), have limited spatial resolution in the mm range, insufficient to resolve cells with the required level of detail^55,56^. On the other hand, while confocal microscopy (CM) provides the required level of detail, the small depth of focus of CM of few hundreds of micrometers renders the technique impractical for applications in tissue engineering and the analysis of 3D models. Thus, many of the difficulties to apply the described protocol to 3D models and xenograft tumors are of technical nature, and can be possibly overcome using more sophisticated methods, such as light-sheet microscopy (LSM)^57,58^ or micro computed tomography. In particular, LSMs are used for in-depth analyses of large, optically cleared samples and long-term three-dimensional (3D) observations of live biological specimens at high spatio-temporal resolution^57,58^. Once that the cells have been located in the 3D region of interest, the relative graphs and topology metrics can be easily derived through few adjustments to the existing algorithm used in this work for 2D architectures. To this aim, in reference^59^ a slightly modified version of the algorithm has been used to examine how neurablastoma cells interact with 3D nanoscale geometries.

The second limitation of this study is that it has been performed on three cell lines of the same type, i.e. lung cancer cells. However, inspired by results of the work, we have designed additional experiments, currently ongoing, to assess the generality of the method. In these extra experiments, we are using a variety of different cell lines, other than lung cancer, to demonstrate that the correlation between the small-world coefficient of cell graphs and the radiation dose is a common characteristic of cancer cells. Preliminary tests showed that H4 epithelial neuroglioma cells, PC3 bone metastasis of grade IV of prostate cancer, and T24 urinary bladder cancer cells exhibit a marked sensitivity to the radiation dose–evidenced by values of small-world-ness that stretch across that 0.5–2 interval for a dose moving from 0 to 6 Gy. The sensitivity of the cells to the dose is also confirmed by morphology analysis of samples: the mean area of cells measured for these sets of the variables increases significantly for increasing values of dose. These data—that have to be confirmed by repeated experiments and will be published upon review elsewhere–illustrate that the topological traits of cell networks and external radiation doses correlate for different types of cells.

In conclusion, this methodology together with the biochemical characterization of the different cancer cells might offer the possibility to shed new light on tumor radiosensitivity and to personalize radiation treatments according to the cancer subtypes, so to reduce the side effects on the healthy tissues and maximize the outcomes of RT.

## Methods

### Cell cultures

Human non-small lung (NCI-H460), and lung carcinoma (A549 and Calu-1) cells were purchased by the ATCC. H460 were cultured in RPMI 1640 medium (Thermo Fischer Scientific), A549 in F12-K medium (Thermo Fischer Scientific) and Calu-1 in McCoy’s medium (Thermo Fischer Scientific). Repeated experiments (data not shown) were performed to determine the cell doubling times in sub-confluent conditions as 20.21 h for the H460, 30.94 h for the A549 and 27.95 h for the Calu-1 cell lines. All cell media were supplemented with Fetal Bovine Serum 10% (Thermo Fischer Scientific) and Pen/Strep 1% (Thermo Fischer Scientific) and maintained at 37 °C in a humidified 5% CO2 atmosphere. All cell lines were authenticated by means of Multiplex human Cell line Authentication (MCA) test. Moreover, all cells were tested for mycoplasma contamination by EZ-PCR Mycoplasma Test Kit (Biological Industries).

### Clonogenic assay and staining

To study the cell topological network after radiation treatment, a classical clonogenic assay experimental setting was used. Briefly, H460, A549 and Calu-1 cells were seeded into six well plates at a density of 100, 150, 300, 1500 and 4000 cells/well after being irradiated with 0, 2, 4, 6, 8 Gy X-rays respectively, with a Multi Rad 225 kV irradiator (Faxitron Biotics). Subsequently, cells were incubated for 6–10 days at 37 °C in a humidified atmosphere with 5% CO2. Due to the different cell-growth rates and exposing doses, the clonogenic assays were stopped between 6 and 10 days to obtain colonies with a desired, similar number of cells, and avoid problems related either to an overgrowth or an excessively low cell number. Following incubation, all samples were fixed in 100% ethanol and stained using a1 µg/ml Hoechst 33342. The plates were stored at 4 °C until imaged.

### Fluorescence imaging

14-bit Images were obtained with an inverted fluorescence microscope (Nikon Eclipse Ti2-E) using a Nikon Plan Fluor 10X or a Nikon S Plan Fluor ELWD 40× objective, a pixel resolution of 0.7373 × 0.7373 µm^2^ or 0.1834 × 0.1834 µm^2^, accordingly, and a Nikon Ds-Qi2 Camera. Excitation of Hoescht was carried out with a Lumencor SOLA II light source and a DAPI filterset.

### Fluorescence image segmentation

More than 2500 images of H460, Calu1 and A549 cells treated at 0, 2, 4, 6, 8 Gy were analyzed with Matlab® (2017b) to extract cell shape descriptors and network parameters^46^. Images were first converted to grayscale and low-pass filtered to remove constant power additive noise before being binarized with Otsu’s method^60^. Morphological opening was performed to remove any small white noises in the image, and morphological closing to remove any small holes in the object. All connected components that had fewer than 8 pixels were removed and structures that were connected to the image border were suppressed. The images were segmented by a watershed transformation^61^ and a distance transform^62^ was used as segmentation function to split out the regions. Watershed transform is known for its tendency to over-segment an image because each local minimum, no matter how small, becomes a “catchment basin”. For this reason, a minima imposition procedure^63^ was implemented: tiny local minima were filtered out and the distance transform was modified so that no minima occurred at the filtered-out locations. With watershed segmentation, single cells were identified. The shape descriptors of the image regions were calculated through the *regionprops* function.

Notice that this procedure represents a first leap towards the automation of conventional clonogenic assay. Performance could be optimized by automatizing image acquisition, which would result in time-saving. The code developed in this study needs hundreds of images to work properly. At the moment, it has a mean absolute elapsed (running) time of $17.65 \pm 2.63$ *s* per image, but it highly depends on the number of nodes of the networks (i.e. the number of cells in the considered image), from which network parameters are calculated. The code is not publically available since it is undergoing modifications and improvements.

Further to this end, one should notice that over the past decades, many computational methods have been proposed to segment cell nuclei, cytoplasm and membranes from 2D microscopy images^64^. Although none of these methods have been explicitly used in radiotherapy. Simple methods, having small computational requirements, include local or global thresholding, based on the histogram of image intensities^65^. Classical approaches as watershed and levelset algorithms^66^ have been used to separate cells resulting overlapped or packed together. Other techniques included segmentation based on mathematical morphology, as active contour models, designed to detect multiple near-circular objects as the cell nuclei^67^. More recently, fully automated methods based on deep neural networks^64^ have been applied for individual cell detection and for differentiating sub-cellular compartments using different fluorescence channels^68^. However, these algorithms are trained on specialized and large datasets, and do not have good performance on different image types, requiring new manual-labelling of data^69^.

### Topological analysis of cell networks

To extract the cell connectivity properties as well as the typology of graph (regular, random or small world) they were forming, we calculated network parameters such as the clustering coefficient (*cc*), the characteristic path length (*cpl*), and the small-world-ness (SW), as described in reference^34^. Briefly, the connections between the nodes were established through the Waxman model^35^, whereby the probability of being a link between two nodes *u* and *v* exponentially decreases with the Euclidean distance *d* between those nodes:

1 $$p\left( {u,v} \right) = \alpha e^{{ - d\left( {u,v} \right)/\beta L}}$$

where *L* is the largest possible Euclidean distance between two nodes of the grid and *α* and *β* the Waxman model parameters that were set to 1 and 0.025, respectively. We decided whether a pair of nodes was connected using the following formula:

2 $$\alpha e^{{ - d_{i,j} /\beta L}} - R \ge 0$$

in which *R* is a constant that we have chosen being 0.1, 0.05, 0.02 so that the probability of being a connection was *p* = 0.9, 0.95, 0.98.

The information about the connections among the nodes of the graph was stored in the *adjacency matrix* $A = a_{ij}$, where the indices *i* and *j* run through the number of nodes *n* in the graph. In the analysis, reciprocity between nodes was assumed, and thus if information could flow from *i* to *j*, it could reversely flow from *j* to *i* (undirected graph).

With these premises, we extracted the local clustering coefficient as

3 $$C_{i} = \frac{{2E_{i} }}{{k\left( {k - 1} \right)}}$$

where *k* is the number of neighbors of a generic node *i*, E_*i*_ is the number of existing connections between those, $k\left( {k - 1} \right)/2$ is the maximum number of connections, or combinations, that can exist among *k* nodes. A global value, *cc*, was derived upon averaging C_*i*_ over all the nodes that composed the graph. Then, the characteristic path length (*cpl*) was determined as the average number of steps along the shortest paths for all possible pairs of network nodes. Once obtained *cc* and *cpl*, such values were compared with those extracted from equivalent Erdos-Rényi (*E–R*) random graphs to determine the small-world coefficient: indeed, a network G with n nodes and m edges is considered small-world if it has a similar path length but greater clustering of nodes than an equivalent Erdos-Rényi (*E–R*) random graph with the same m and n^36^.

### Statistical analysis

Results in the article and in the figures are reported as mean ± standard deviation. We used a Student’s t-test statistics (two-tailed, unpaired) to perform comparison between means of different groups, where we assumed that elements in each group are normally distributed. In performing the test, the null hypothesis is that the means between pairs of samples are equal. Everywhere in the text and the figures the difference between two subsets of data is considered statistically significant if the Student’s t-test gives a significant level *p* (*p* value) less than 0.05.

## Supplementary Information

Supplementary Information.

## Data Availability

Research data and related metadata are available upon reasonable request to the corresponding author.
