# Supplementary material for: Human lung-cancer-cell radioresistance investigated through 2D network topology
Source: Sci Rep. 2022 Jul 28;12:12980. doi: 10.1038/s41598-022-17018-0 (PMC9334295; doi:10.1038/s41598-022-17018-0)
Supplement: Supplementary file 1 — Supplementary Information. [file 41598_2022_17018_MOESM1_ESM.docx]

**Human lung-cancer-cell radioresistance investigated through 2D network topology**

Luca Tirinato ^1, 2,^ *, Valentina Onesto ^1,^ *, Daniel Garcia-Calderon ^2, 3^ , Francesca Pagliari ^2^, Maria-Francesca Spadea ^4^, Joao Seco ^2, 3^ †, Francesco Gentile ^1,^ †

^1^ Nanotechnology Research Center, Department of Experimental and Clinical Medicine, University of Magna Graecia, Catanzaro, 88100, Italy

^2^ Biomedical physics in radiation oncology, DKFZ German cancer research center, Heidelberg, Germany

^3^ Department of physics and astronomy, Heidelberg university, Heidelberg, Germany

^4^ Department of Experimental and Clinical Medicine, University of Magna Graecia, Catanzaro, 88100, Italy

* These authors contributed equally to this work

† Corresponding Authors: [j.seco@dkfz-heidelberg.de](mailto:j.seco@dkfz-heidelberg.de), [francesco.gentile@unicz.it](mailto:francesco.gentile@unicz.it)

**Supporting Information**

| Networks associated to the different cell lines (of H460, A549 and Calu-1) used in this study, upon exposure to a dose in the $0-8$ Gy range. | Pg. 2 |
| --- | --- |
| High magnification fluorescent images of H460, A549 and Calu-1 cells taken at different values of the irradiation dose, i.e. $0$, $2$, $8$ Gy. | Pg. 17 |
| SW coefficients of the of H460, A549 and Calu-1 cell networks, determined at different values of the Waxman cutoff probability: $p=0.9$ and $p=0.98$. | Pg. 18 |
| The p-values measuring the difference between the SW coefficients determined for the cell networks built using different values of the Waxman cutoff probability: $p=0.9$ and $p=0.95$. | Pg. 18 |


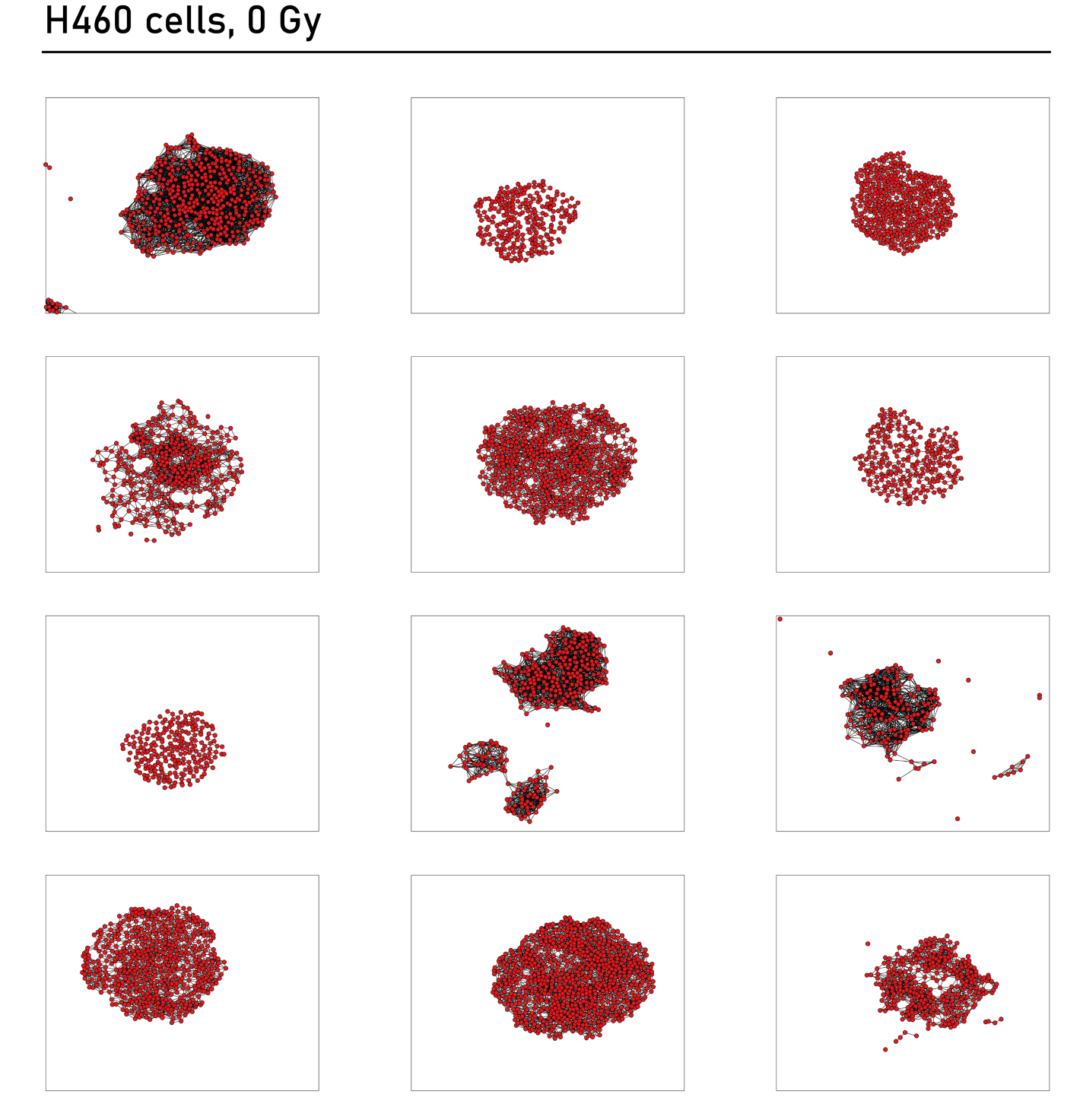


**Supporting Information Figure 1.1.** ***Networks associated to the H460 cells*.** The image reports examples of cell-graphs derived from H460 cells without external radiation treatment.


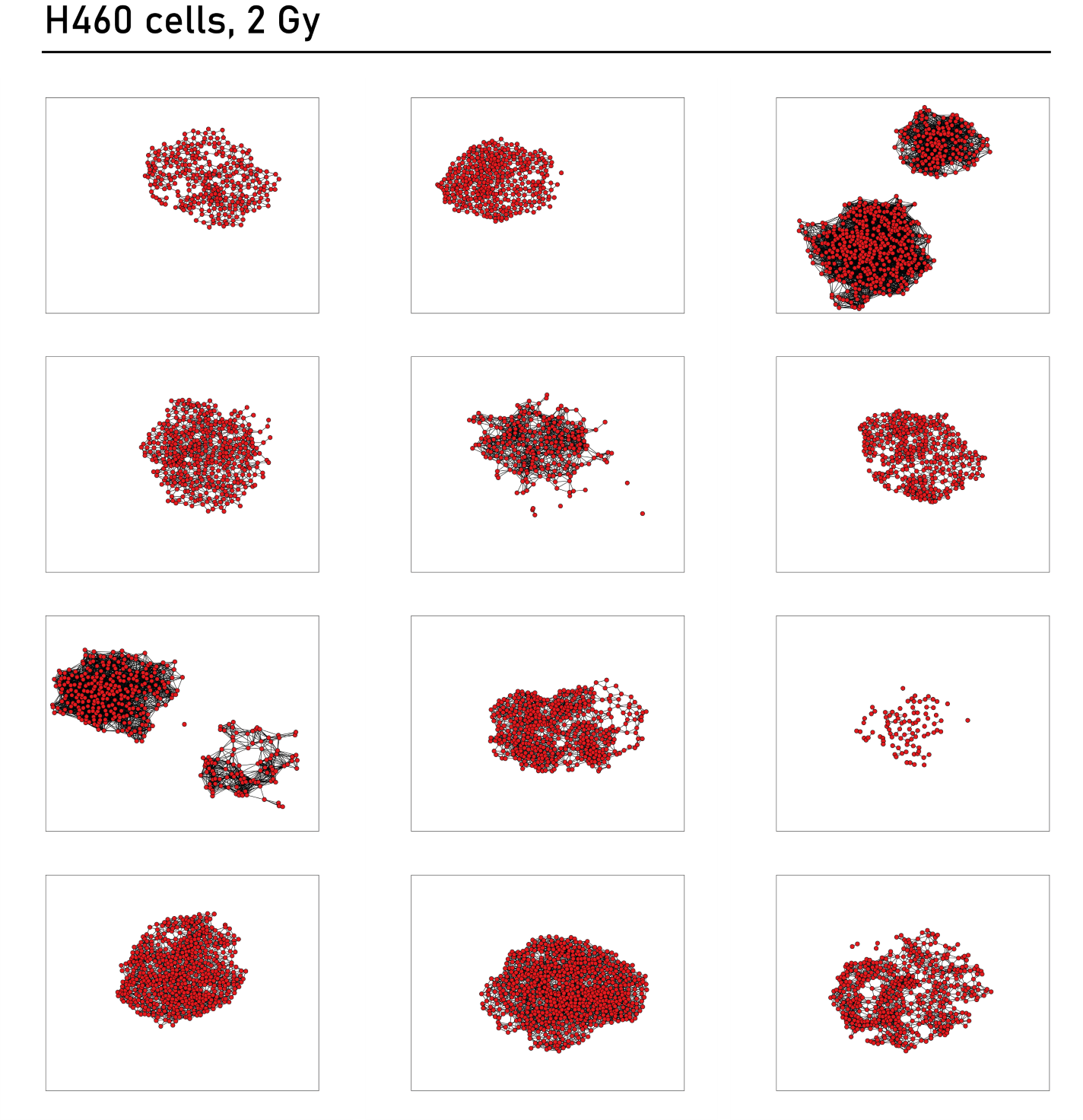


**Supporting Information Figure 1.2.** ***Networks associated to the H460 cells upon exposure to a*** $\boldsymbol{2}$ ***Gy dose*.** The image reports examples of cell-graphs derived from H460 cells after exposition to an external ionizing radiation of 2 Gy.


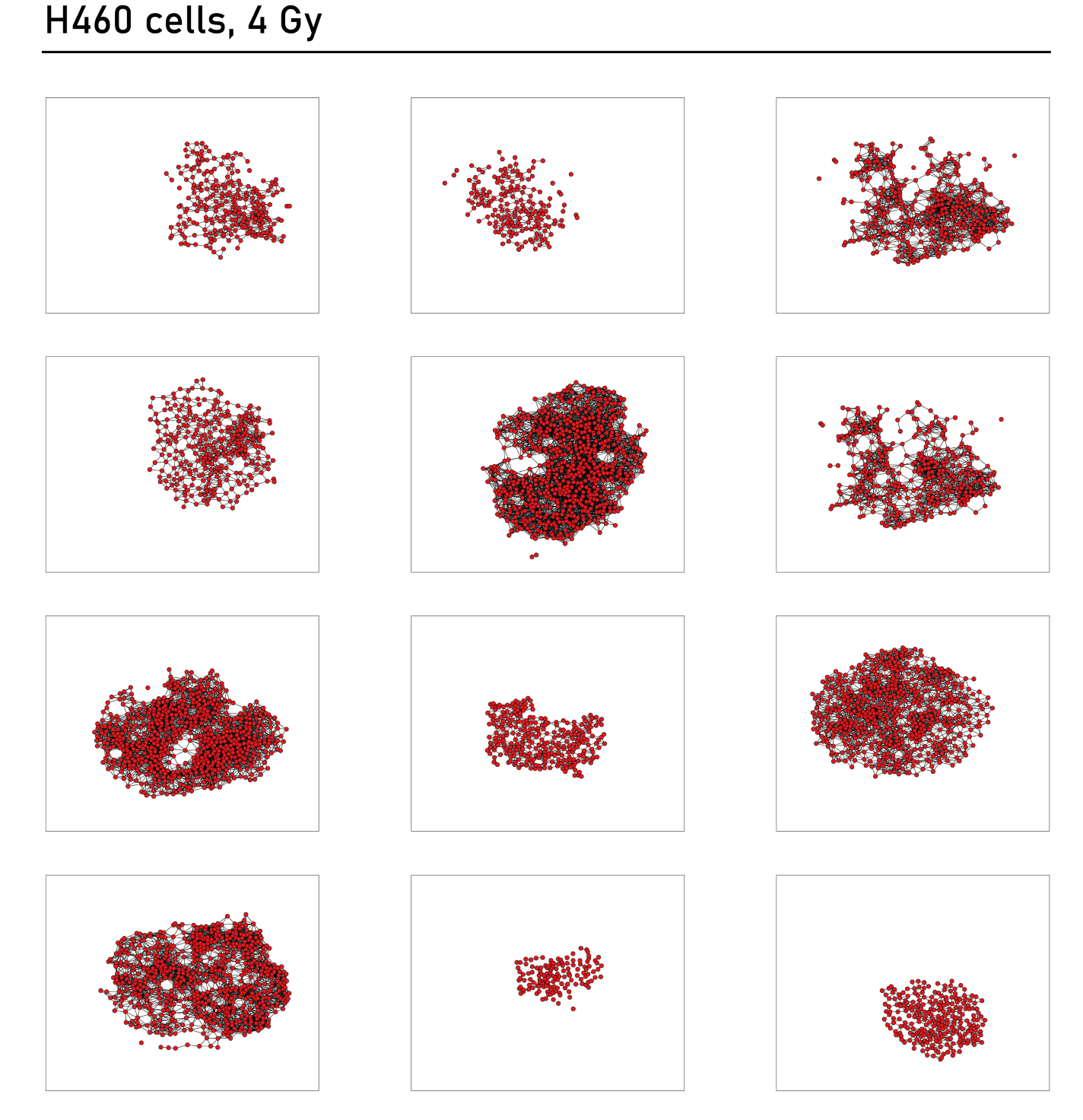


**Supporting Information Figure 1.3.** ***Networks associated to the H460 cells upon exposure to a*** $\boldsymbol{4}$ ***Gy dose*.** The image reports examples of cell-graphs derived from H460 cells after exposition to an external ionizing radiation of 4 Gy.


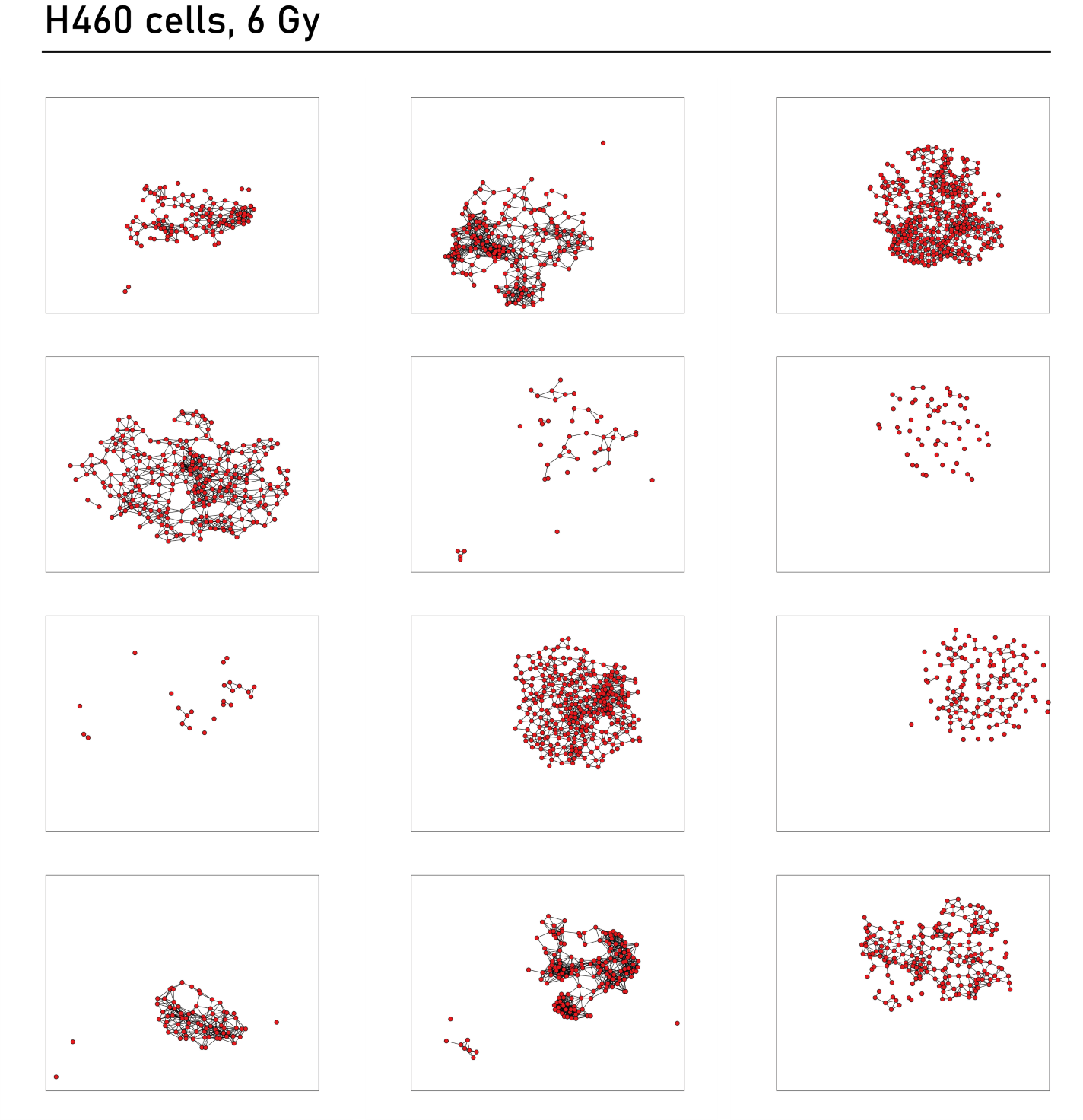


**Supporting Information Figure 1.4.** ***Networks associated to the H460 cells upon exposure to a*** $\boldsymbol{6}$ ***Gy dose*.** The image reports examples of cell-graphs derived from H460 cells after exposition to an external ionizing radiation of 6 Gy.


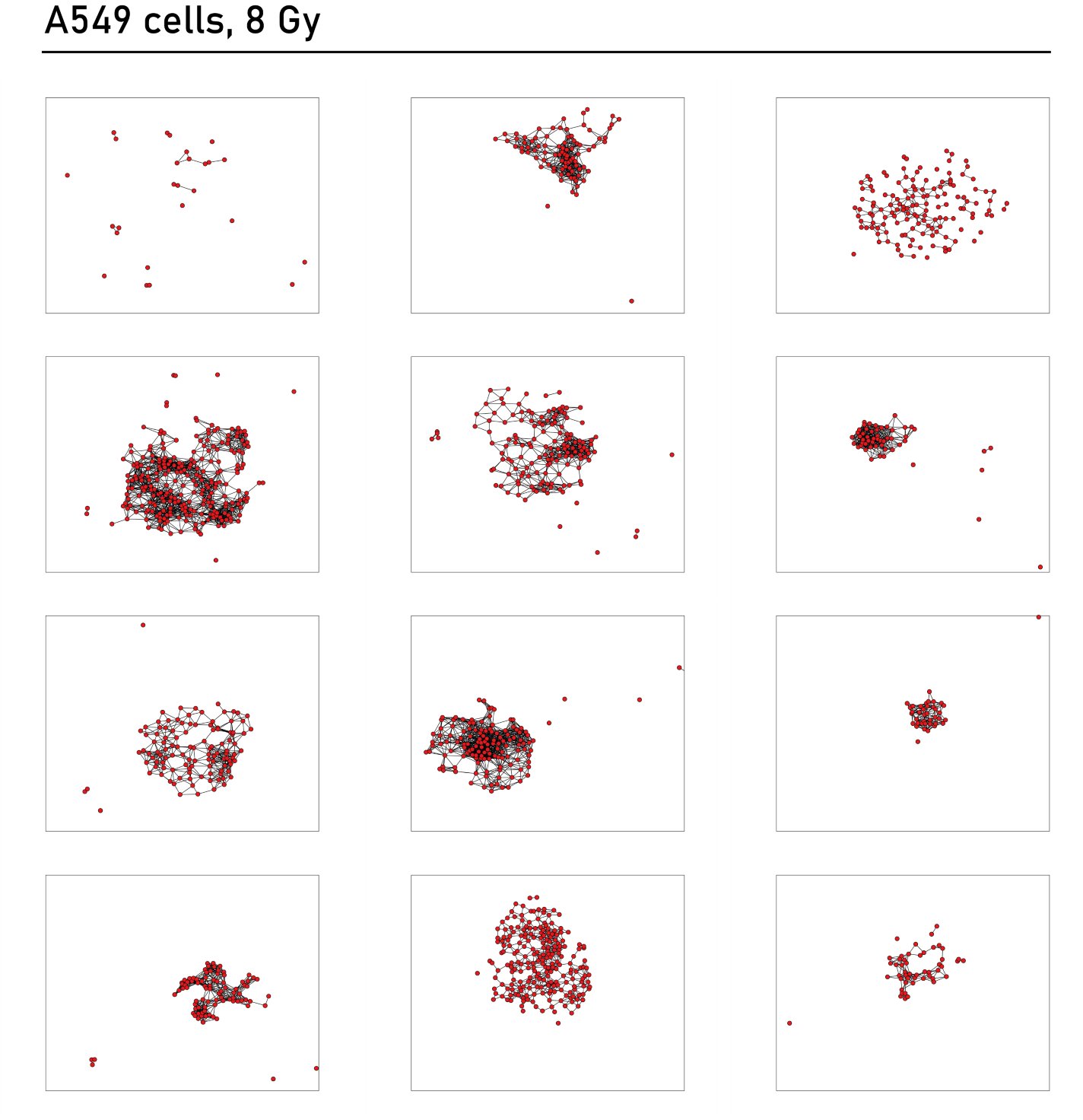


**Supporting Information Figure 1.5.** ***Networks associated to the H460 cells upon exposure to a*** $\boldsymbol{8}$ ***Gy dose*.** The image reports examples of cell-graphs derived from H460 cells after exposition to an external ionizing radiation of 8 Gy.


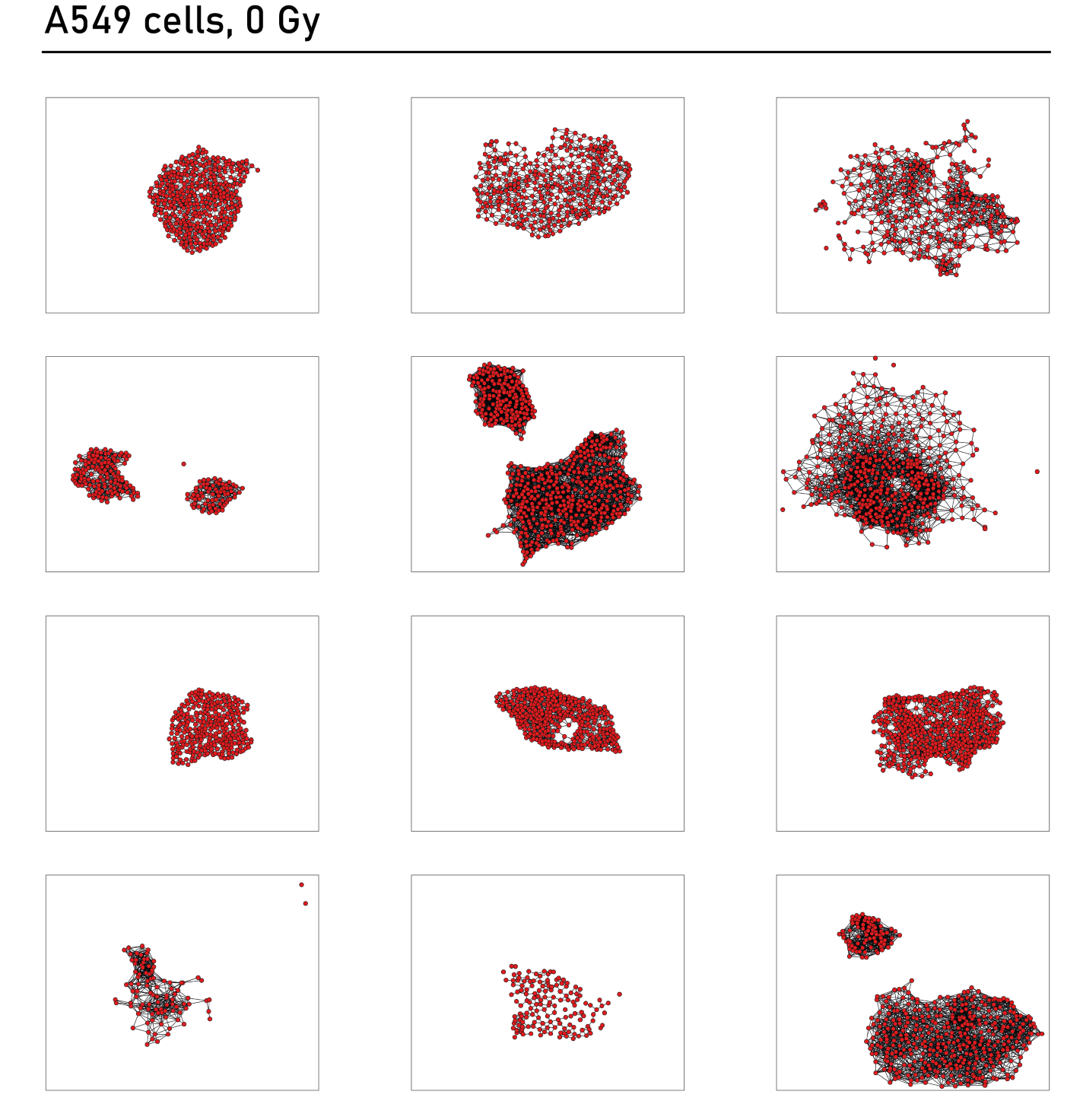


**Supporting Information Figure 1.6.** ***Networks associated to the A549*.** The image reports examples of cell-graphs derived from A549 cells without external radiation treatment.


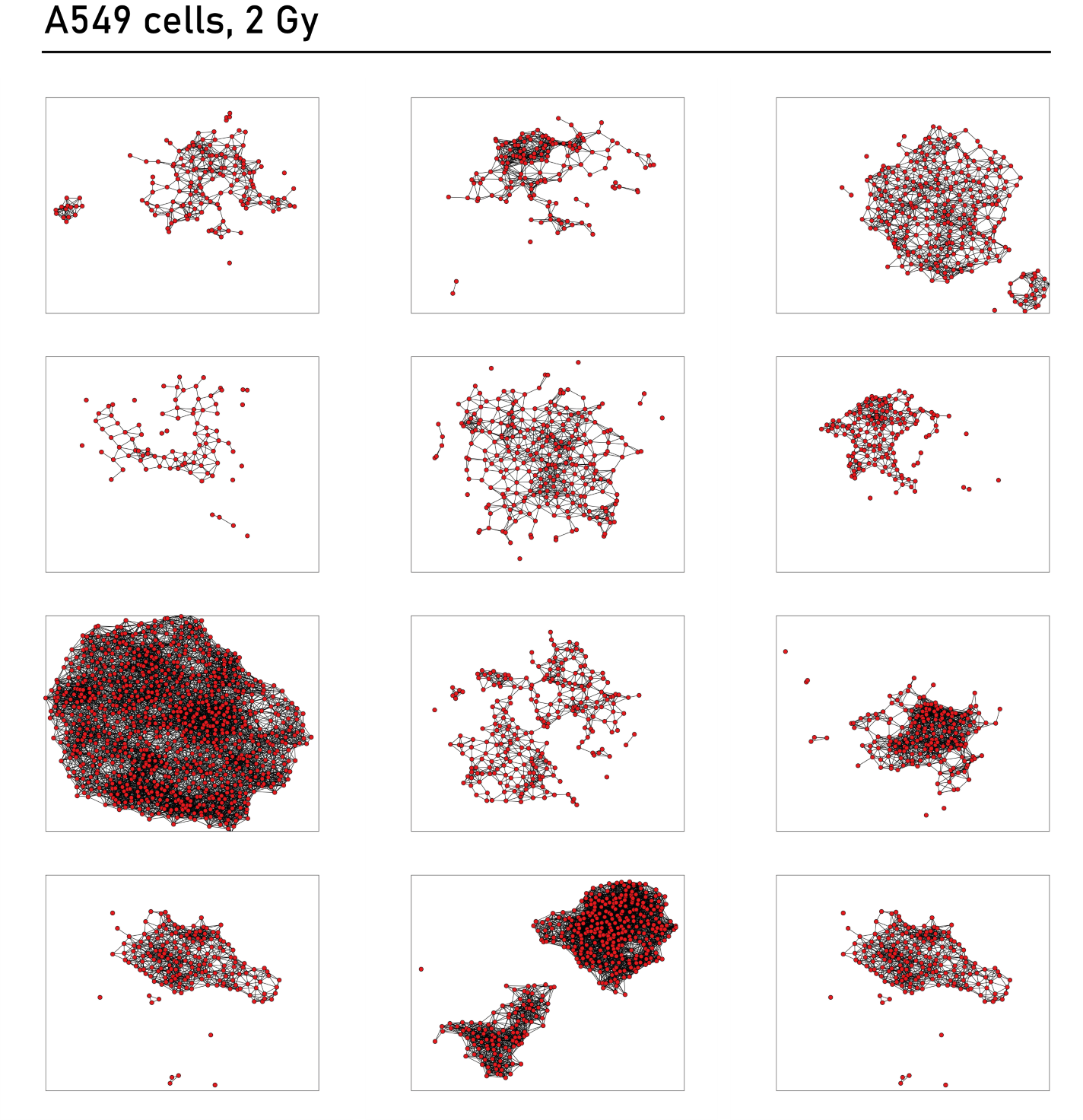


**Supporting Information Figure 1.7.** ***Networks associated to the A549 cells upon exposure to a*** $\boldsymbol{2}$ ***Gy dose*.** The image reports examples of cell-graphs derived from A549 cells after exposition to an external ionizing radiation of 2 Gy.


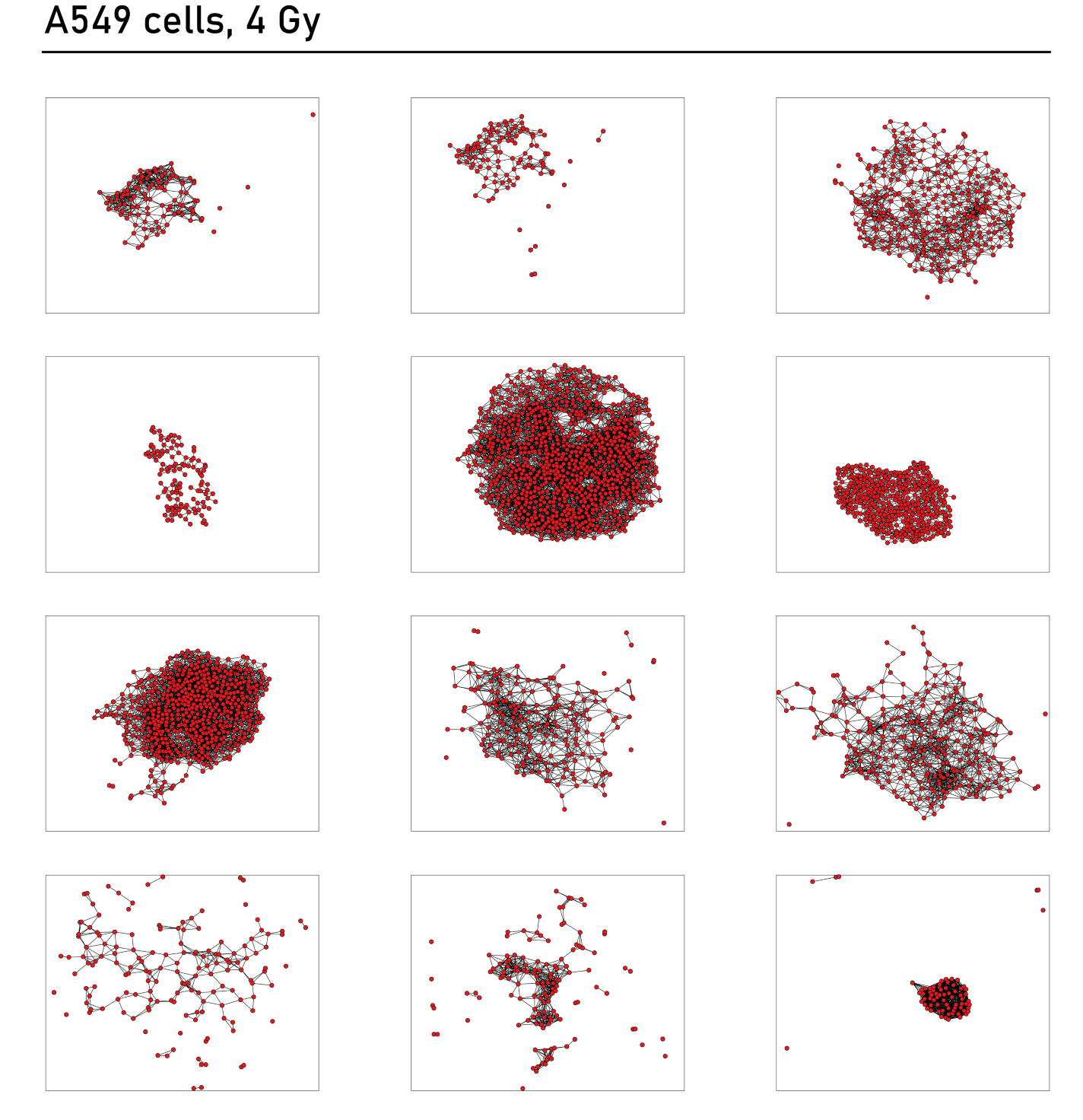


**Supporting Information Figure 1.8.** ***Networks associated to the A549 cells upon exposure to a*** $\boldsymbol{4}$ ***Gy dose*.** The image reports examples of cell-graphs derived from A549 cells after exposition to an external ionizing radiation of 4 Gy.


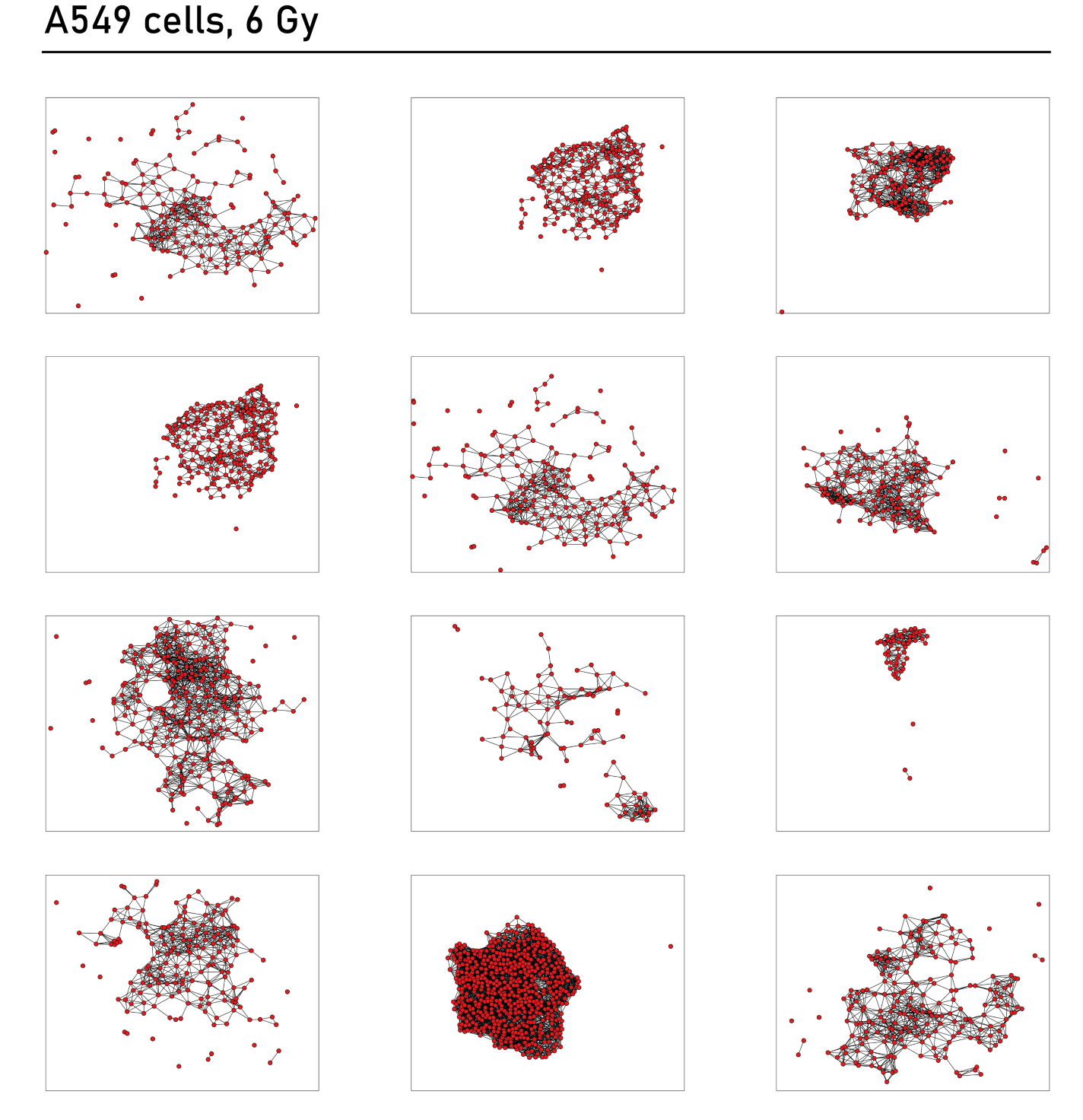


**Supporting Information Figure 1.9.** ***Networks associated to the A549 cells upon exposure to a*** $\boldsymbol{6}$ ***Gy dose*.** The image reports examples of cell-graphs derived from A549 cells after exposition to an external ionizing radiation of 6 Gy.


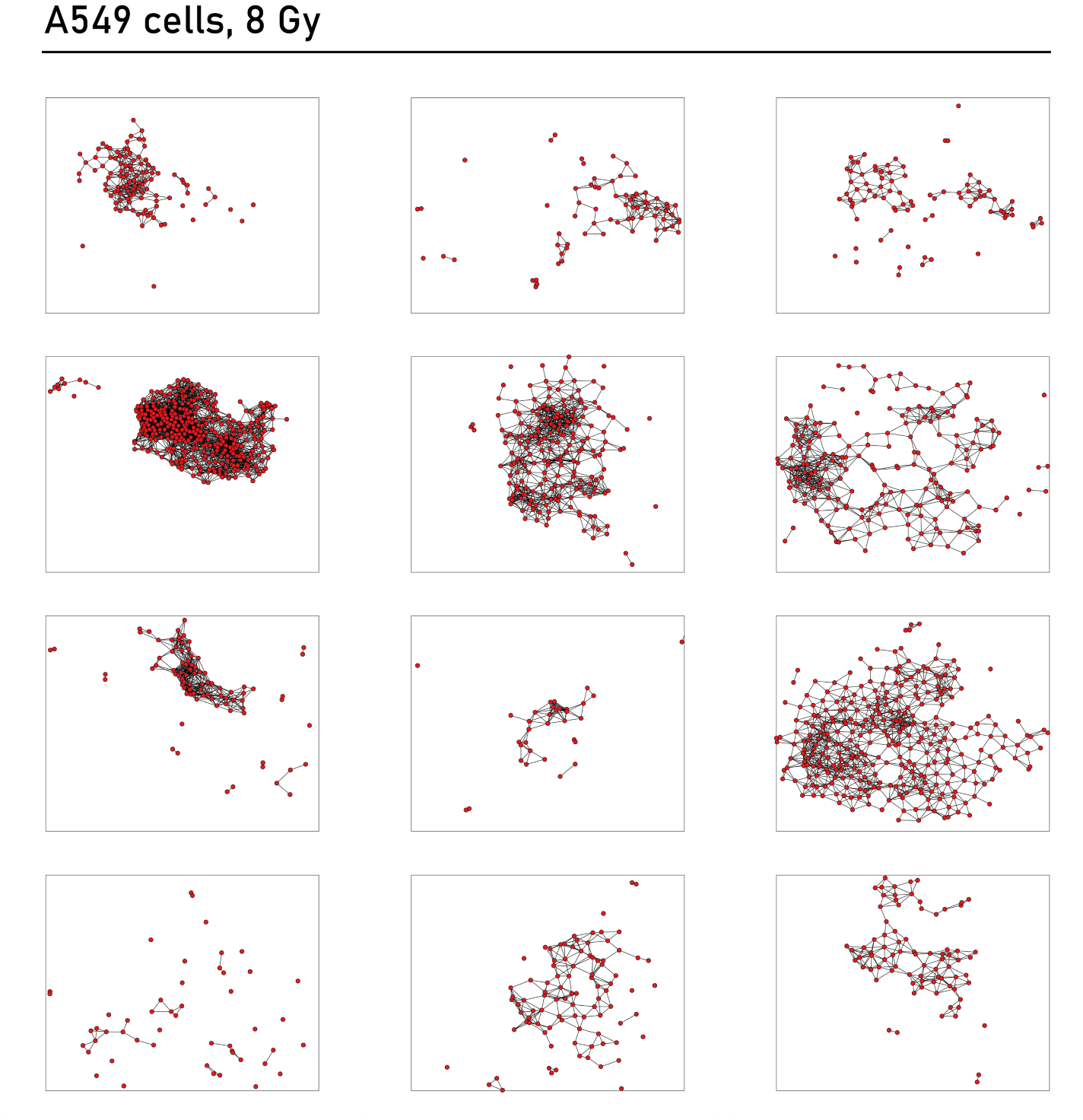


**Supporting Information Figure 1.10.** ***Networks associated to the A549 cells upon exposure to a*** $\boldsymbol{8}$ ***Gy dose*.** The image reports examples of cell-graphs derived from A549 cells after exposition to an external ionizing radiation of 8 Gy.


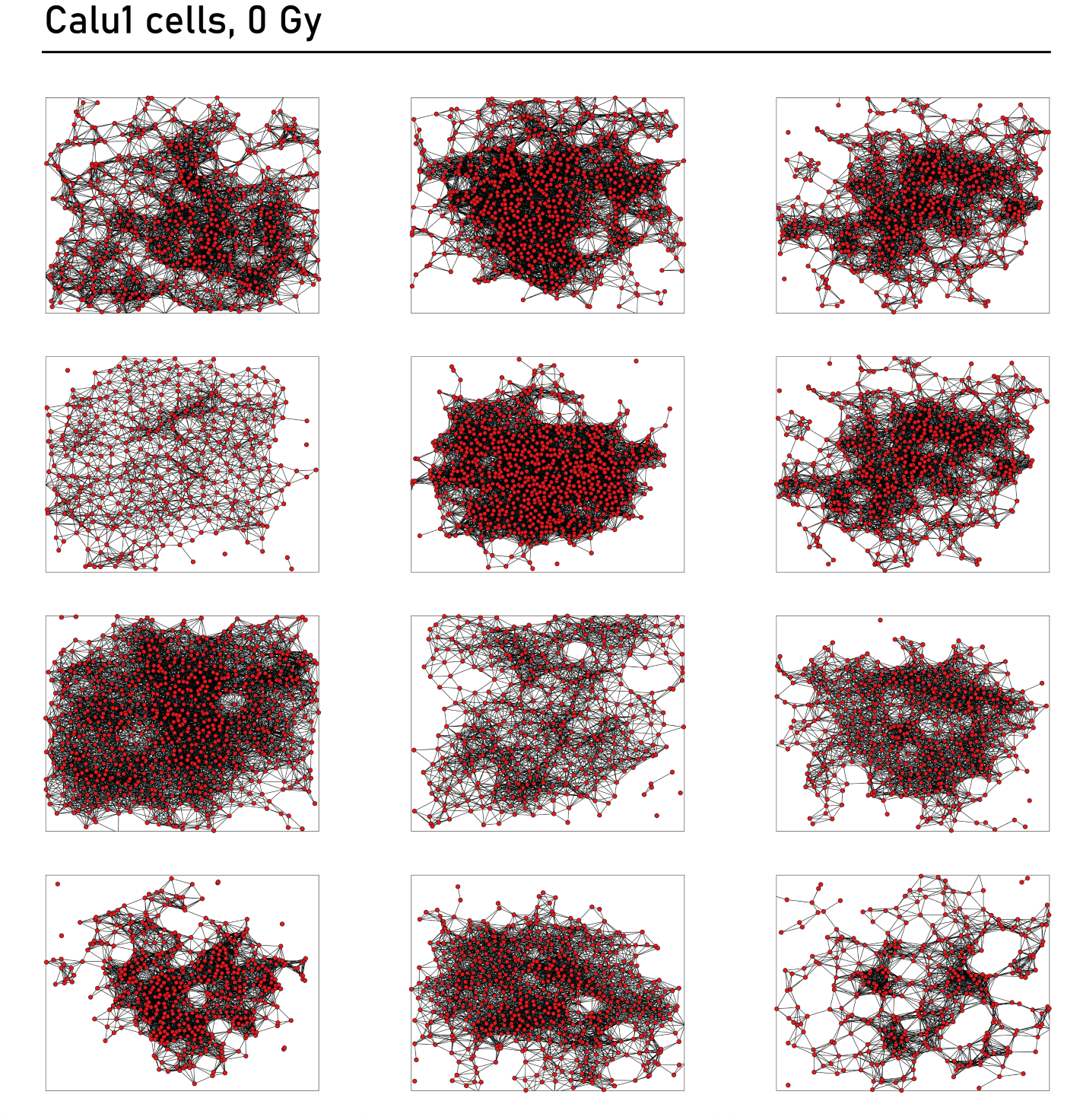


**Supporting Information Figure 1.11.** ***Networks associated to the Calu1 cells*.** The image reports examples of cell-graphs derived from Calu1 cells without external radiation treatment.


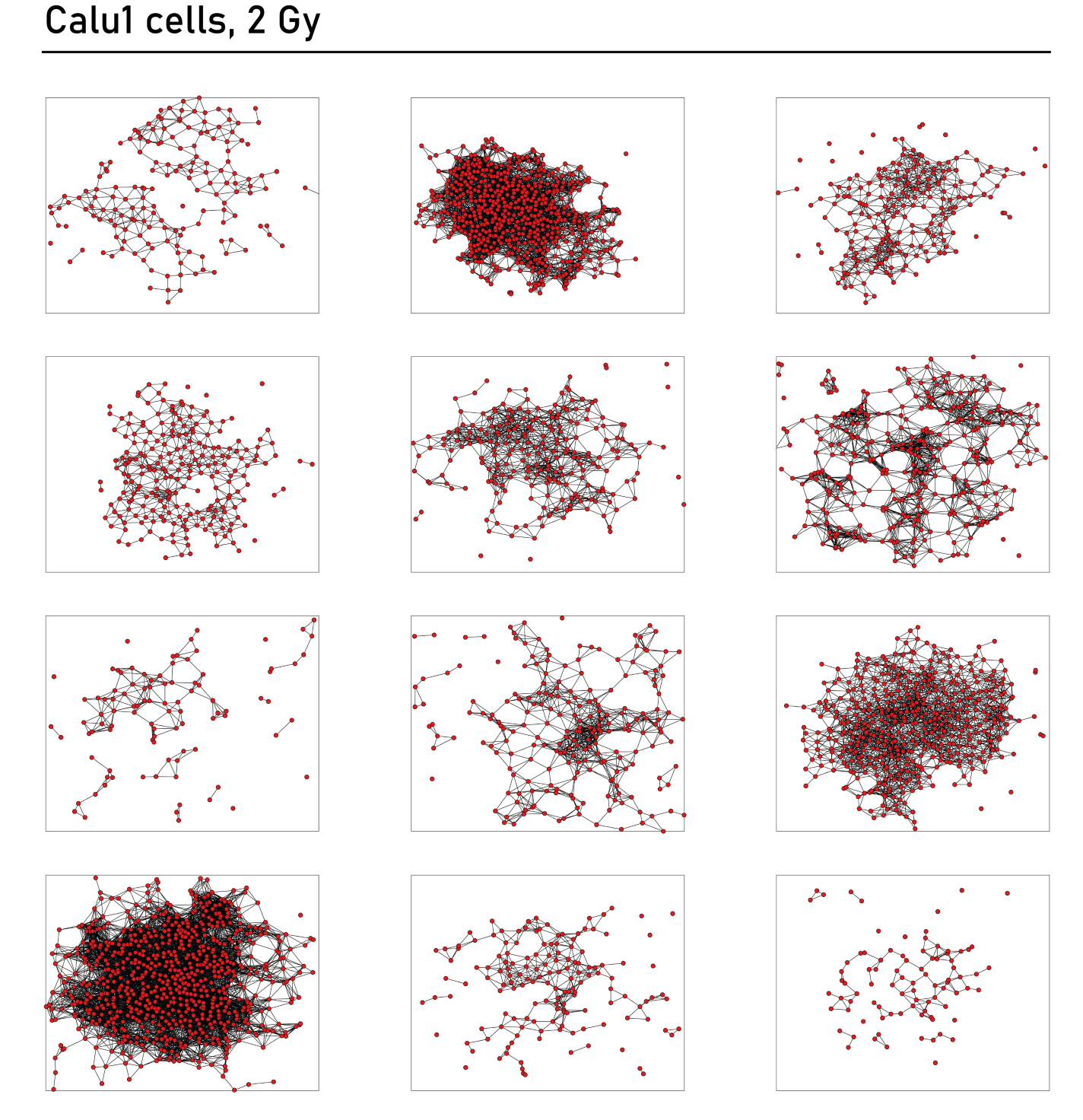


**Supporting Information Figure 1.12.** ***Networks associated to the Calu1 cells upon exposure to a*** $\boldsymbol{2}$ ***Gy dose*.** The image reports examples of cell-graphs derived from Calu1 cells after exposition to an external ionizing radiation of 2 Gy.


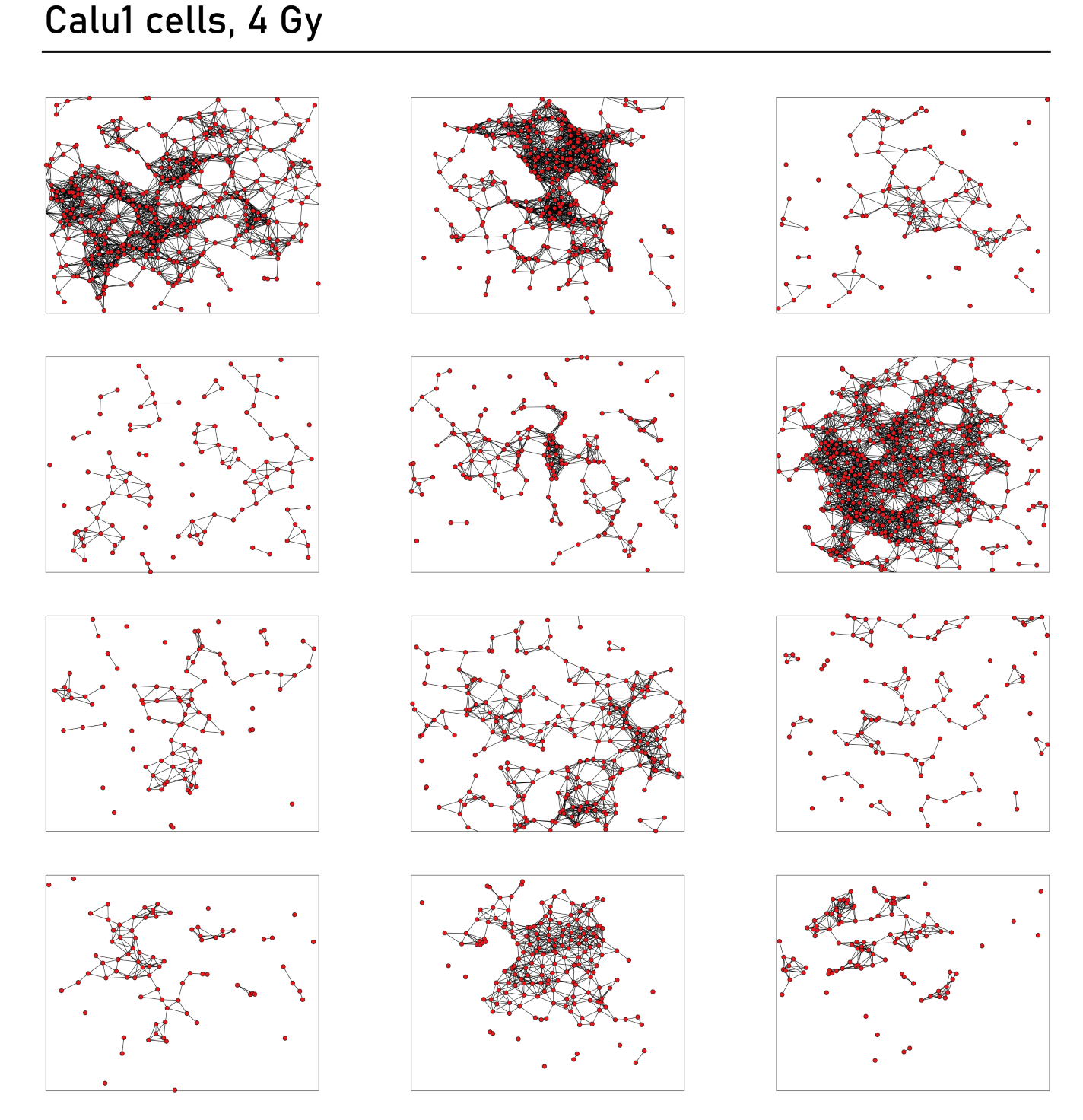


**Supporting Information Figure 1.13.** ***Networks associated to the Calu1 cells upon exposure to a*** $\boldsymbol{4}$ ***Gy dose*.** The image reports examples of cell-graphs derived from Calu1 cells after exposition to an external ionizing radiation of 4 Gy.


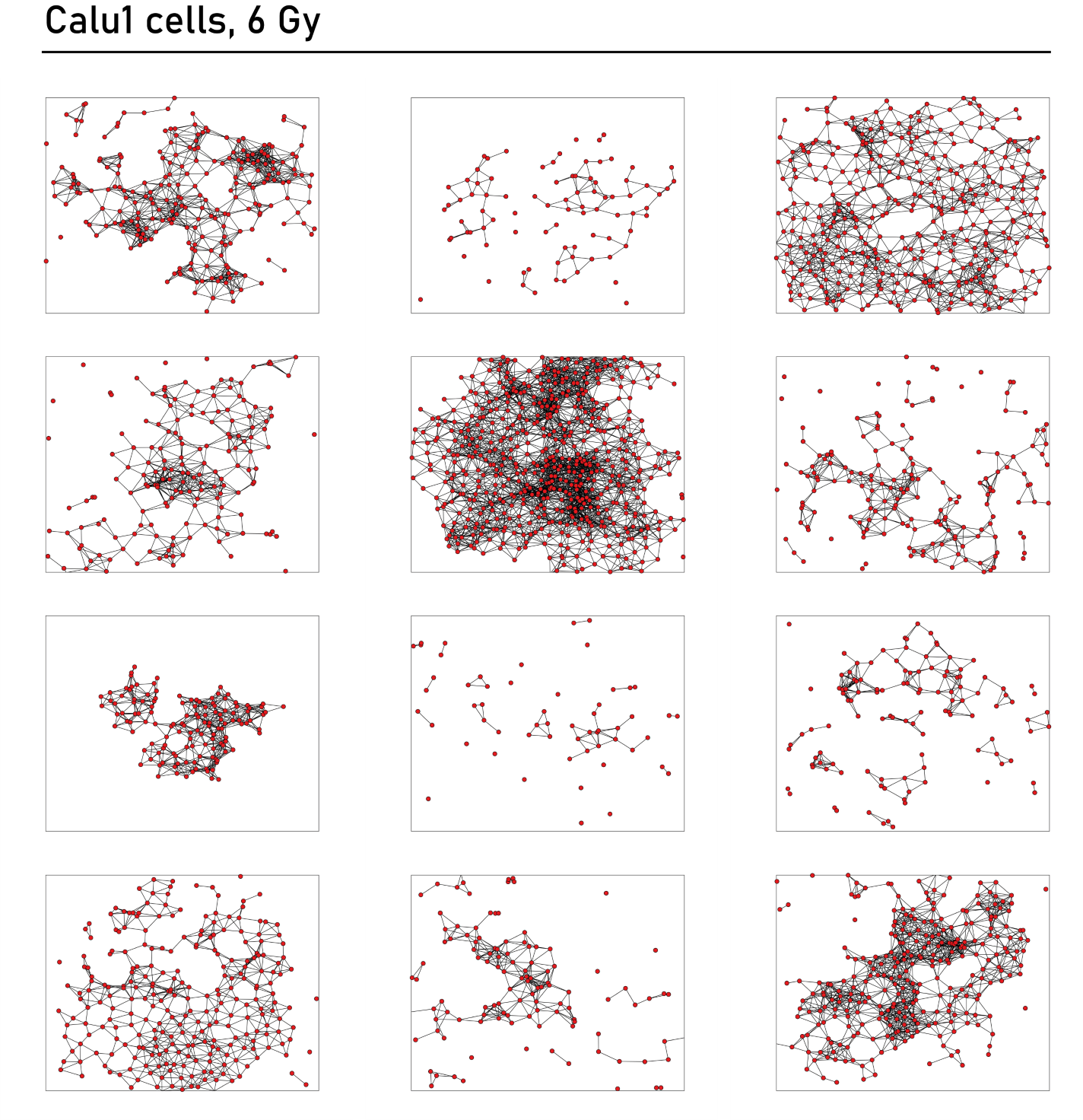


**Supporting Information Figure 1.14.** ***Networks associated to the Calu1 cells upon exposure to a*** $\boldsymbol{6}$ ***Gy dose*.** The image reports examples of cell-graphs derived from Calu1 cells after exposition to an external ionizing radiation of 6 Gy.


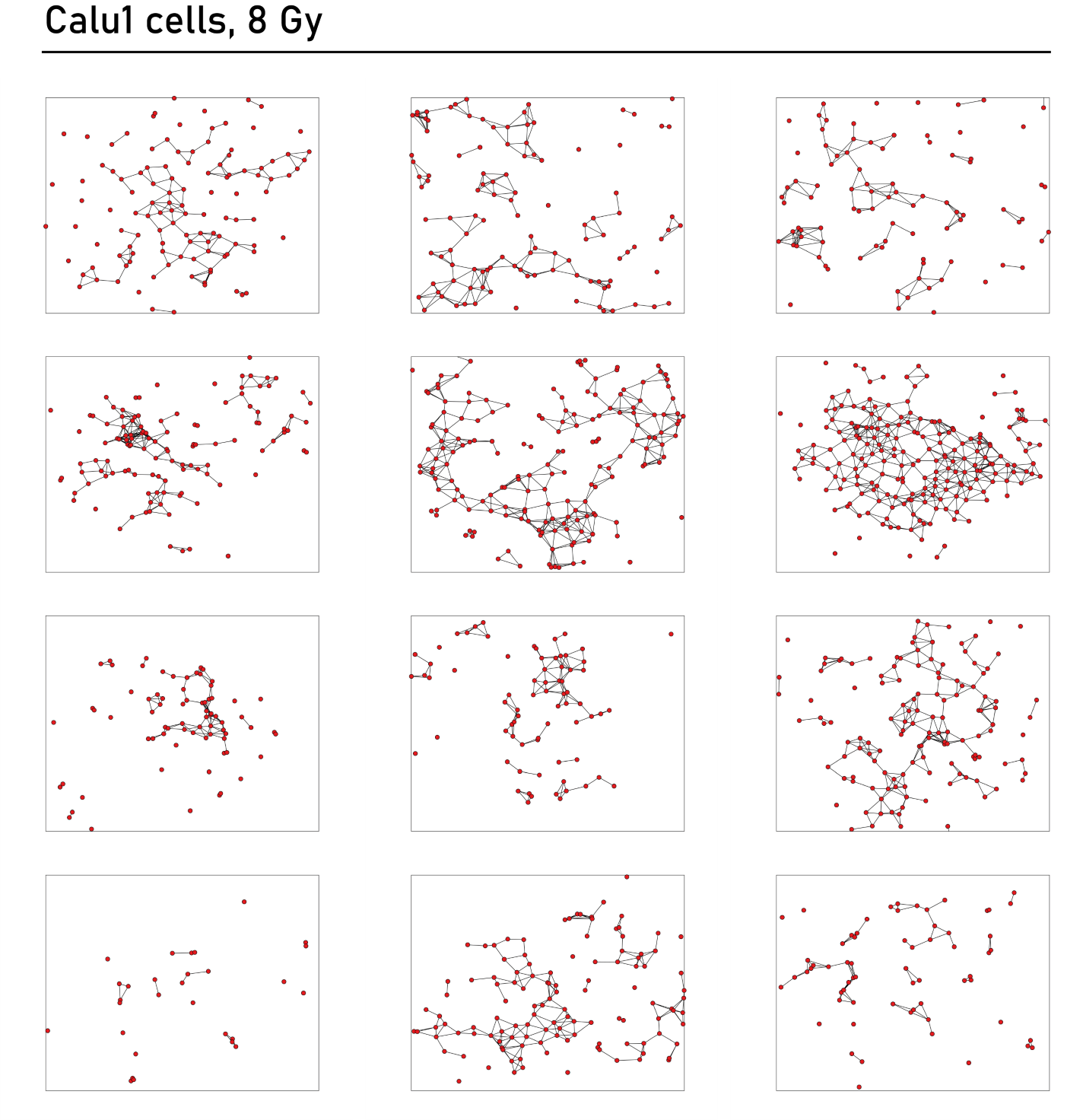


**Supporting Information Figure 1.15.** ***Networks associated to the Calu1 cells upon exposure to a*** $\boldsymbol{8}$ ***Gy dose*.** The image reports examples of cell-graphs derived from Calu1 cells after exposition to an external ionizing radiation of 8 Gy.


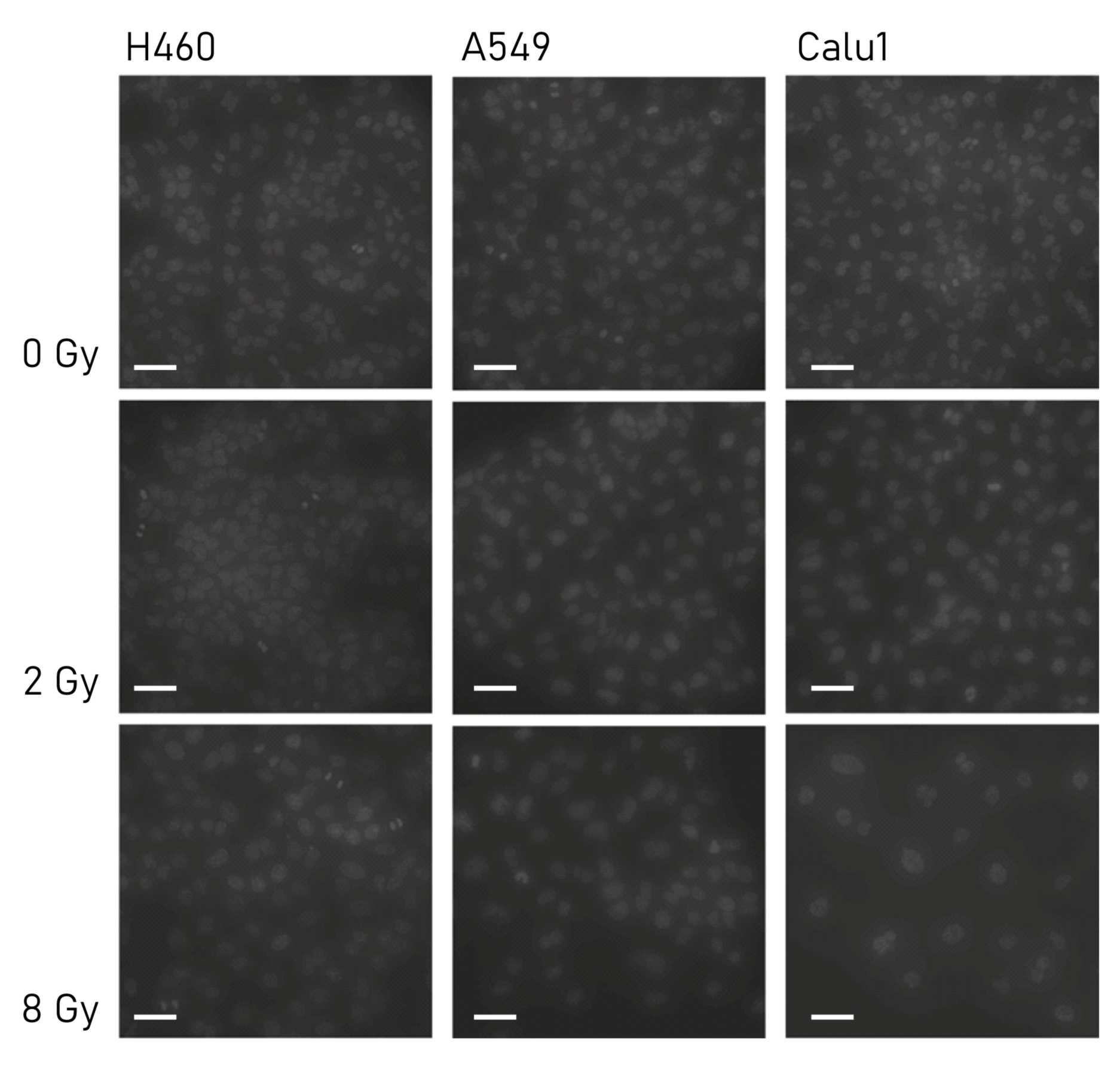


**Supporting Information Figure 2.** ***High-magnification fluorescence images of lung-cancer-cells upon radiation treatment*.** 40x high magnification representative fluorescent images of H460, A549 and Calu-1 cells taken at different values of the irradiation dose, i.e. $0$, $2$, $8$ Gy. The images show that cell morphology is influenced to some extent by the external radiation. The scale bar reported for all images is $100 \mu m$.


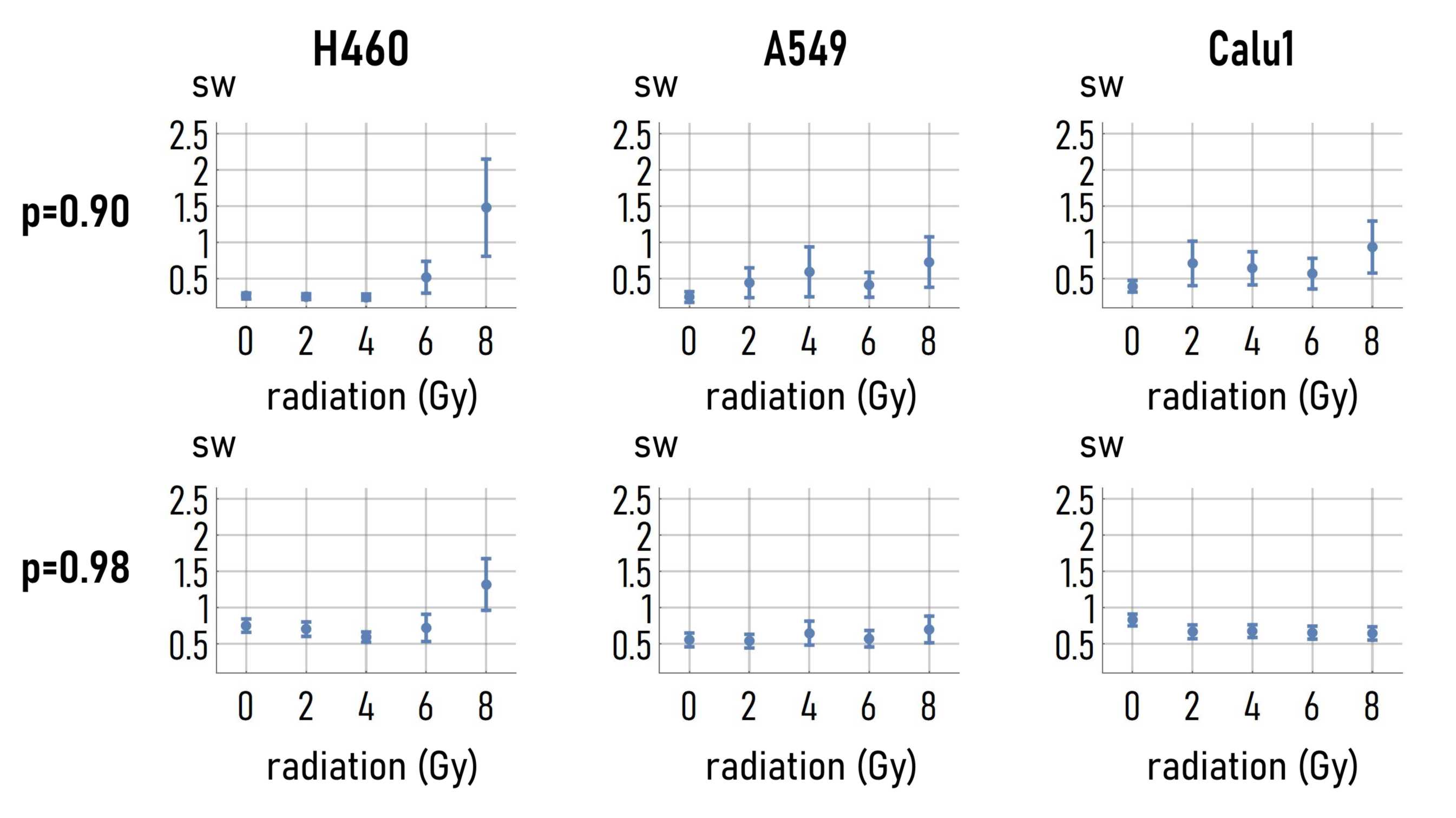


**Supporting Information Figure 3a. *Topological metrics of lung-cancer-cell graphs*.** Small-world coefficient of lung-cancer-cell graphs as a function of the external radiation dose, determined for different values of the Waxman’s wiring probability threshold $p$ – the analysis was here performed for the H460, A549 and Calu1 cell-lines.


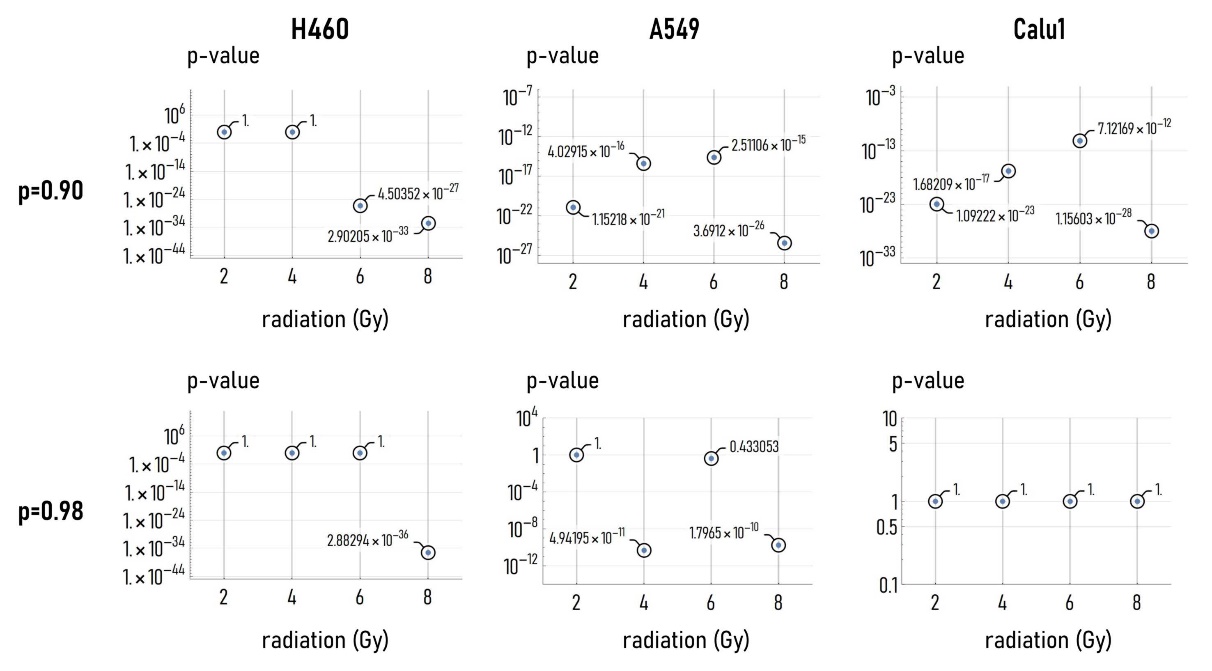


**Supporting Information Figure 3b. *Statistical analysis of the small-word coefficient distribution as a function of the external radiation dose*.** The diagrams report the p-value resulting from a Student’s T test statistics performed on the small-world coefficients of the cell graphs, as a function of the radiation dose and the Waxman’s wiring probability threshold $p$. Small values of p-value indicate that the difference between the considered case and the 0-dose reference level, is statistically significative.
